# Lentivector cryptic splicing mediates increase in CD34+ clones expressing truncated *HMGA2* in human X-linked severe combined immunodeficiency

**DOI:** 10.1038/s41467-022-31344-x

**Published:** 2022-06-28

**Authors:** Suk See De Ravin, Siyuan Liu, Colin L. Sweeney, Julie Brault, Narda Whiting-Theobald, Michelle Ma, Taylor Liu, Uimook Choi, Janet Lee, Sandra Anaya O’Brien, Priscilla Quackenbush, Tyra Estwick, Anita Karra, Ethan Docking, Nana Kwatemaa, Shuang Guo, Ling Su, Zhonghe Sun, Sheng Zhou, Jennifer Puck, Morton J. Cowan, Luigi D. Notarangelo, Elizabeth Kang, Harry L. Malech, Xiaolin Wu

**Affiliations:** 1 Laboratory of Clinical Immunology and Microbiology, NIAID, NIH, Bethesda, MD 20892 USA; 2 Cancer Research Technology Program, Leidos Biomedical Research, Inc., Frederick National Laboratory for Cancer Research, Frederick, MD 21702 USA; 3 Experimental Cell Therapeutics Lab, St. Jude Children’s Research Hospital, Memphis, TN 38105 USA; 4 Division of Allergy Immunology and Blood and Marrow Transplantation, Department of Pediatrics, University of California San Francisco and UCSF Benioff Children’s Hospital, San Francisco, CA 94143 USA

**Keywords:** Transcriptional regulatory elements, Translational research

## Abstract

X-linked Severe Combined Immunodeficiency (SCID-X1) due to *IL2RG* mutations is potentially fatal in infancy where ‘emergency’ life-saving stem cell transplant may only achieve incomplete immune reconstitution following transplant. Salvage therapy SCID-X1 patients over 2 years old (NCT01306019) is a non-randomized, open-label, phase I/II clinical trial for administration of lentiviral-transduced autologous hematopoietic stem cells following busulfan (6 mg/kg total) conditioning. The primary and secondary objectives assess efficacy in restoring immunity and safety by vector insertion site analysis (VISA). In this ongoing study (19 patients treated), we report VISA in blood lineages from first eight treated patients with longer follow up found a > 60-fold increase in frequency of forward-orientated VIS within intron 3 of the High Mobility Group AT-hook 2 gene. All eight patients demonstrated emergence of dominant *HMGA2* VIS clones in progenitor and myeloid lineages, but without disturbance of hematopoiesis. Our molecular analysis demonstrated a cryptic splice site within the chicken β-globin hypersensitivity 4 insulator element in the vector generating truncated mRNA transcripts from many transcriptionally active gene containing forward-oriented intronic vector insert. A two base-pair change at the splice site within the lentiviral vector eliminated splicing activity while retaining vector functional capability. This highlights the importance of functional analysis of lentivectors for cryptic splicing for preclinical safety assessment and a redesign of clinical vectors to improve safety.

## Abstract (web summary)

De Ravin et al. report an unplanned interim analysis of a secondary safety outcome for an ongoing clinical trial on lentiviral gene therapy for the treatment of X-linked Severe Combined Immunodeficiency (NCT01306019). Vector induced alternative splicing events are identified that cause aberrant fusion transcripts, leading to clonal dominance in a single patient and clonal expansion in others. This can be mitigated by the removal of the lentivector cryptic splice acceptor.

## Introduction

Ex vivo lentivector-mediated transduction of autologous CD34+ hematopoietic stem cells (HSC) as gene therapy has achieved significant clinical benefit for many inherited diseases^1–5^. Some clinical trials using murine gamma retrovirus vectors for autologous HSC gene therapy resulted in malignancy and myelodysplasia from insertional activation of nearby oncogenes from the action of enhancer elements within the vector long terminal repeat (LTR)^6^. Until recently, vector integration mediated oncogenesis has not been observed in any of the many hundreds of patients treated with self-inactivating (SIN) lentivector-transduced autologous HSCs over the past decade. The observed lack of vector integration mediated oncogenesis by SIN lentivectors is presumed to be due to the introduction of the U3 deletion of enhancer elements in the LTR that confers the SIN characteristic, plus a genome insertional pattern for lentivectors that is less likely to affect nearby gene promoter activation compared to murine gamma retrovirus vectors. However, the recent case of leukemia in a patient with adrenoleukodystrophy (ALD) treated with SIN lentivector (bluebird bio) demonstrated a breakthrough of these safety barriers when a strong internal promoter like MND is used^7^. This further highlights the importance of long-term genetic analysis of lineage specific vector integration site (VIS) patterns for safety assessment of patients treated with lentivector gene therapy.

Surveillance for potential genotoxicities relies on characterization of the VIS landscape across blood lineages. Observing a diverse polyclonal pattern, lack of emergence of increasing dominance of a blood lineage by a single clone, and lack of significant progressive disturbance of hematopoietic lineage cell proportions are generally considered indications of safety. Since 2012, we have been conducting a clinical trial of SIN lentivector transduced autologous HSC for older children and young adults with X-linked severe combined immune deficiency (SCID-X1) who entered this clinical trial because haploidentical transplant with no or minimal conditioning in infancy had resulted in incomplete immune reconstitution (ClinicalTrials.gov Identifier: NCT01306019). The patients at entry into our study had donor engraftment in only the T lymphocyte lineage, required supplemental IgG, and had increasing medical problems including in most cases chronic norovirus infection. In 2016 we reported clinical outcomes for the first five patients, demonstrating significant restoration of T cell, B cell and NK cell immunity in all patients. The first two patients (both young adults) at >2 years’ follow up had achieved independence from IgG supplementation, had mounted protective responses to immunization, and had eradicated their long-standing chronic norovirus infection^3^. In that report, we observed in those first two patients that there was an increasing over-representation of VIS within the High Mobility Group AT-hook 2 gene (*HMGA2*) relative to the otherwise abundant diverse VIS in other genes and intergenic regions. We noted no disturbance in hematopoiesis pattern, no expansion of any lineage beyond the normal range and no emergence of leukemic clones. By 2016, a total of 8 patients had been treated with clinical benefit from the gene therapy for all but Subject P8 reproducing the improvement in immune function together with the clinical benefit reported for the first five patients. However, more recently we noted in Subject P6 the appearance of a single clonal population containing a forward-oriented insertion in intron 3 of *HMGA2* comprising 15–22% of the CD34+ hematopoietic progenitor and myeloid lineages without any evidence for disturbance in hematopoiesis or any cytogenetic abnormalities. The molecular mechanism for this clonal dominance in Subject P6 and the relative increase in VIS within *HMGA2* in the other patients is the basis of this report.

Here, we show transcriptional profiling of peripheral blood immune cells and single-cell colony analysis of CD34+ HSCs and inducible pluripotent stem cells (iPSCs) reveal vector-induced alternative splicing events to create aberrant fusion transcripts that lead to the clonal dominance in P6 and clonal expansions in other patients. We confirm that the potent gene-trapping effect of the vector can be mitigated by the removal of its cryptic splice acceptor (SA). This report show results of an unplanned interim analysis of a secondary safety outcome.

## Results

### Long-term polyclonal multi-lineage gene marking in LVSCID-X1

Lentivector gene therapy was used to treat older patients with SCID-X1 who continued to experience significant medical problems due to persistent immune deficiency despite prior haploidentical hematopoietic cell transplant without myeloid conditioning that had conferred only limited engraftment of T cell progenitors. We have treated 15 subjects in this study to date (the first eight subjects between 2012 and 2016; the second 7 subjects treated since 2019), but will only present detailed analysis of the first eight subjects that allowed an extensive evaluation of the long-term kinetics of VIS patterns (Table 1).

**Table 1:** Patient characteristics and summary of unique integration sites.

	P1	P2	P3	P4	P5	P6	P7	P8
*IL2RG* mutation	c. 823 T > G	c447 del A	c.923 C > A	c.341 G > A	c 31 T > A	c.938 + 1 G > A	c694G > A	c.31 T > A
Age at treatment (years)	23	22	7	16	10	23	3	12
VISA follow up (months) (2^0^)	84	24	54	48	54	48	42	38
VCN (infusion) (1^0^)*	0.27	0.17	0.31	0.57	0.36	0.41	0.3	0.27
VCN Myeloid (2 yr) (1^0^)*	0.079	0.13	0.0768	0.47	0.61	1.76	0.03	0.07
VCN T (2 yr) (1^0^)	0.12	0.57	0.14	0.84	1.57	0.58	0.97	0.04
VCN B (2 yr) (1^0^)*	0.38	0.36	0.27	0.94	1.07	0.92	0.14	0.13
VCN Natural killer (2 yr) (1^0^)*	0.77	0.56	0.52	2.33	2.01	4.46	0.29	0.34
1^0^ baseline clinical problems	Waning T, B dysfunction, low wt	Waning T, B dysfunction, low wt, chronic lung disease	Waning T, B dysfunction, FTT Infections	Waning T, B dysfunction, FTT Infections	Waning T, B dysfunction, FTT	Waning T, B dysfunction, low wt, infections	Donor CD8 T infiltration, hepatocellular injury	Waning T, B dysfunction, FTT, infections
IgG supplement (1^0^)	Off	Off	Off	Off	Off	Off	freq	On
Noro (1^0^)	N/A	X	Cleared	Cleared	Cleared	Cleared	N/A	persists
Albumin (1^0^)	normalized	normalized	normalized	normalized	normalized	normalized	normalized	normalized
Skin-warts, molluscum (1^0^)	Same	Same	Same	Same	Same	Same	Same	Same
Lungs (1^0^)	well	Fatal pulm hemorrhage	Well	Well	Well	Well	Well	Well
Diarrhea (1^0^)	N/A	N/A	Cleared	Cleared	Cleared	Cleared	N/A	Same
Total unique IS**	116,660	55,354	97,543	495,223	589,147	479,035	44,213	198,214
Unique IS in B cell (2^0^)**	73,816	26,041	51,620	256,327	248,447	185,693	21,462	36,193
Unique IS in T cell (2^0^)**	14,628	3863	15,208	88,605	96,946	31,681	12,390	13,951
Unique IS in NK cell (2^0^)**	19,142	8779	9590	115,598	98,103	79,869	10,290	47,208
Unique IS in polymorphonuclear cells (2^0^)**	29,947	10,762	36,275	122,735	211,781	157,350	11,266	82,961
Unique IS in CD14+ (2^0^)**	25,289	10,343	32,174	110,785	181,202	141,994	10,590	76,722

Table 1 summarizes patient-specific characteristics and the cumulative unique integration sites (IS) for each lineage. Since unique IS are frequently shared among multiple lineages, the sum of IS in each lineage differs from the total unique IS in each patient. Unique IS are not shared among different patients. The cumulative IS in P2 is prematurely truncated due to a fatal pulmonary hemorrhage at 2.5 years after treatment. P8 was retreated at 30 months and only IS from first treatment is included. NA indicates Not applicable and N for normal. *VISA* vector integration site analysis, *IS* insert site, *VCN* vector copy number, *FTT* failure to thrive, yr year, wt weight. (1^0^) and (2^0^) indicates primary and secondary objectives of the clinical study respectively. *Copies per cell; **Number of unique sites per ug genomic DNA.

**Fig. 1:**
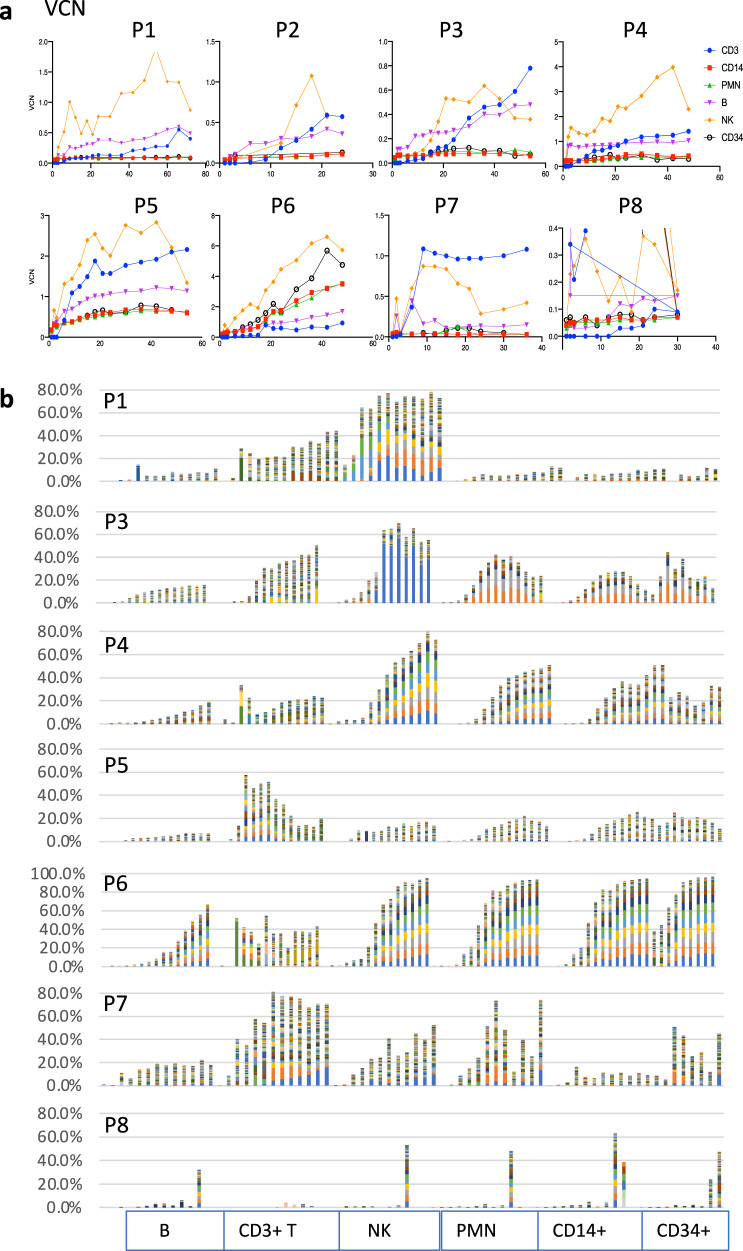
Chronological surveillance of gene marking and polyclonal vector insertion sites (VIS) in sorted immune cells following lentivector (LV) gene therapy in X-SCID patients (*n* = 8). **a** Surveillance of multi-lineage gene marking in sorted immune (CD3, CD14, PMN, B, NK, and CD34+) cell lineages over time following gene therapy. **b** Tracking of vector integration sites at different time points in sorted immune cell lineages for each patient treated with LV-gene therapy. Bar-chart shows top 256 VIS in each patient. Low-frequency clones not shown. Source data for all patient vector integration sites and vector copy number are provided as Source Data files.

Stable multi-lineage gene marking was observed following gene therapy in all of the first 8 patients treated in this study (Fig. 1a). As expected, there was substantial increase in vector copy number (VCN) in T, B, and NK cell lineages over time because of the physiologic growth advantage provided by the *IL2RG* transgene-mediated production of common gamma chain protein (IL2Rγc) essential to the restoration of function of the receptors for Interleukins 2, 4, 7, 9, 15 and 21^8–12^. However, there was also a slight to modest increase in VCN in myeloid lineages and peripheral blood circulating CD34+ progenitors. In general, the greatest increase in VCN was observed in NK cells, often exceeding 1 copy/cell (e.g., P1, 4, 5, and 6), suggesting a selection for higher copy number of the *IL2RG* transgene during the reconstitution of NK cells in these patients. Subject P2 had longstanding very severe bronchiectasis pre-gene therapy and died from a fatal pulmonary bleed at 2.5 years after treatment; and Subject P8 did not achieve levels of gene marking sufficient to provide clinical benefit and was successfully retreated with gene therapy at 30 months. For this reason, data for P2 and P8 (Table 1) are truncated at 24 months and 30 months, respectively.

Extensive VIS analysis was performed over time and across lineages for all eight patients allowing assessment of over 2 million unique vector integration sites from the eight patients during the 4–8 year follow-up period tracked (Fig. 1B, Table 1, Fig. S2). This formed the basis for assessing the relative frequency of inserts within individual genes that allowed detection of an increased frequency of VIS in *HMGA2*.

### HMGA2 VIS are enriched in all patients

**Fig. 2:**
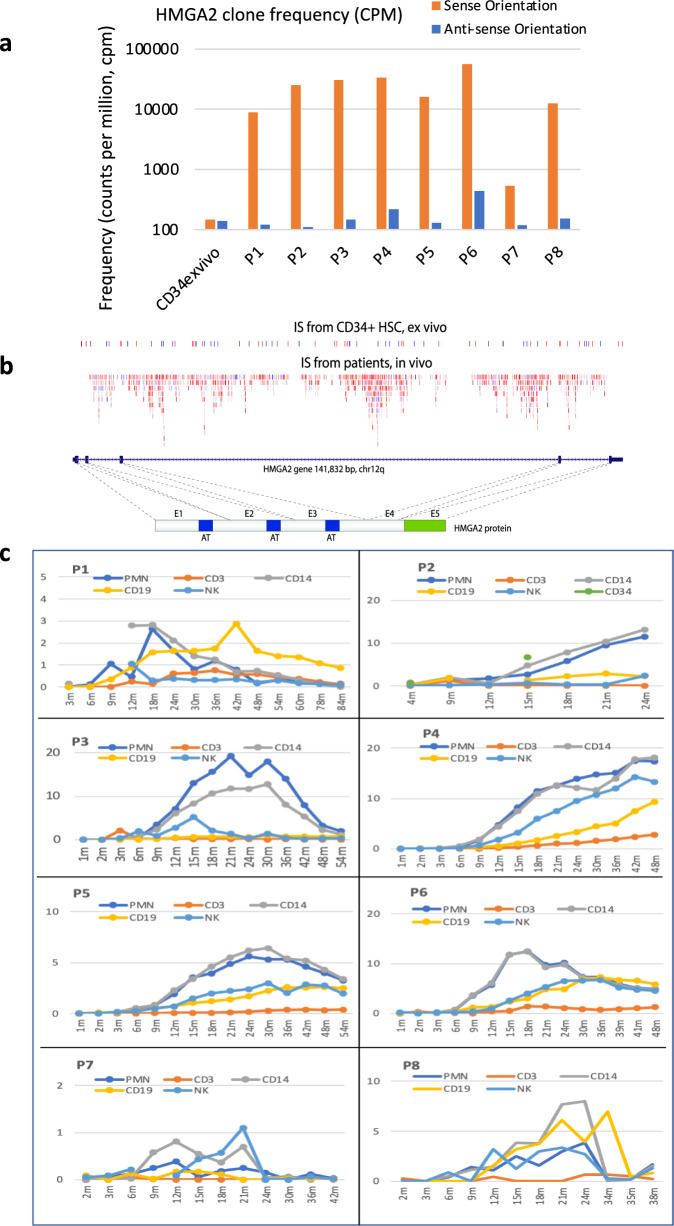
In vivo enrichment of HMGA2 clones with vector insertion in sense orientation and tracking of the highly enriched HMGA2 clones in all patients following LV-gene therapy. **a** Unique *HMGA2* VIS containing clones are detected at higher frequency and *HMGA2* VIS represent a greater proportion of all inserts detected from all patient samples compared to baseline ex vivo transduced CD34+ HSC product data sets. Shown is the frequency of detection of *HMGA2* VIS calculated from cumulative VIS data for each patient including all time points and cell lineages, and then normalized as counts per million total VIS detected. Frequencies were calculated separately for both orientations of *HMGA2* VIS provirus (Forward/Sense versus Reverse/Anti-Sense orientation as the *HMGA2* gene). The in vivo frequency is much higher than that observed from the ex vivo transduced CD34+ HSC product for sense orientation, but not for antisense orientation. Note the *Y*-axis is log10 scale. **b** Shown is the distribution and frequency of unique Vector IS across the HMGA2 gene as detected in the ex vivo transduced CD34+ HSC product data sets (upper row) compared to the in vivo patient blood lineage derived data sets (second row). The blue or red colors, respectively, denote each unique *HMGA2* VIS containing clone where provirus is in the sense orientation (blue) or antisense orientation (red) of the gene. The darker the color shading the larger the unique clone. Most insertion sites are in intron 3 of HMGA2 gene, which separates the Hmga2 protein to the N-terminal AT-hook DNA-binding domain and C-terminal acidic domain. c *HMGA2* VIS clone dynamics in vivo by peripheral blood lineage in each Subject. *HMGA2* VIS clone frequency was calculated as percent of all VIS at each time point in different lineages. In general, the highest frequency of *HMGA2* VIS clones is seen in PMN, and CD14 cells, but also can be seen in other lineages like NK and CD19 cells. Note the different scale for the *y*-axis (percentage of total VIS).

We compared the cumulative incidence of VIS in *HMGA2* from peripheral blood lineages with the baseline incidence of *HMGA2* VIS in the infusion product in vitro (Fig. 2a). For this analysis we distinguished between *HMGA2* VIS in the sense (forward) orientation versus the antisense (reverse) orientation relative to the *HMGA2* coding sequence. The frequency of forward and reverse orientation *HMGA2* VIS were approximately equal in the baseline transduced CD34+ product and were at a frequency similar to that expected for a gene of this size. However, there was a significant increase of the in vivo frequency of *HMGA2* VIS in all patients, specifically for VIS in the sense orientation as the *HMGA2* coding sequence (Fig. 2a), suggesting a cis-element(s) in the vector contributing to a selective clonal expansion. Most of the VIS landed in introns 2, 3, 4 of *HMGA2*, with the largest intron 3 harboring 99% of these forward-oriented VIS (Fig. 2A, B). Next, our VIS analysis in individual peripheral blood (PB) bead-selected myeloid (CD14+ , polymorphonuclear (PMN)) and lymphoid (CD3+ T, CD19+ B, and NK) cells showed the highest frequency of *HMGA2* VIS clones in myeloid cells (especially in P2, 3, 4, 5, 6), reaching ~20% of all VIS (Fig. 2C). Of note, the frequency declined in all patients (except P4) after ~2 years following treatment with no changes in their immune cell numbers, indicating that while *HMGA2* VIS clones have a growth advantage over other clones, overall lineage cell production remained under homeostatic regulation.

### Truncated *HMGA2* transcript causes expansion of CD34^+^*HMGA2* VIS clones

**Fig. 3:**
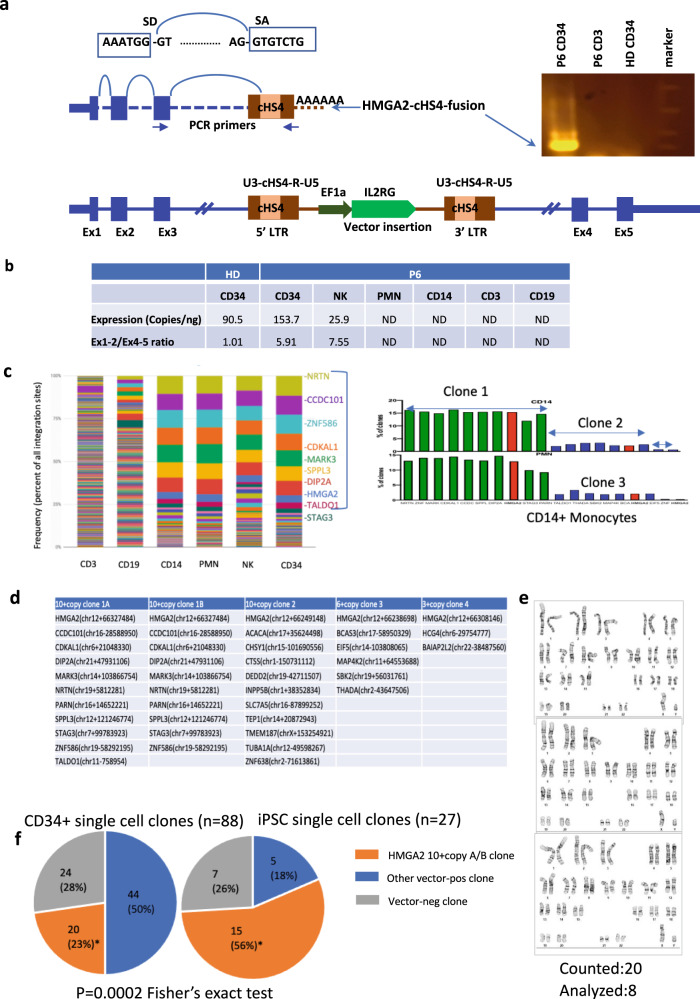
Characterization of *HMGA2*-cHS4 fusion transcripts in single-cell CD34+ clones and derived-iPSCs clones with potential synergy in multi-insert clones. **a** Illustration of lentivector insertion in the 3^rd^ intron of HMGA2 gene in the bottom of the panel. Fusion transcript was generated by *HMGA2* exon1-exon2-exon3 and spliced into the cHS4 SA in the 5LTR of the vector. Splicing Donor (SD) of HMGA2 exon3 and splicing acceptor (SA) of cHS4 sequences are shown on the top. PCR primers used to amplify the fusion transcript from P6 CD34 cells are located on *HMGA2* exon3 and vector LTR. Amplified fusion transcript from RTPCR is shown in the right. **b** Quantification of *HMGA2* transcript in different blood lineages in P6 and the 5′/3′ ratio, both determined by ddPCR as described in methods. *HMGA*2 is expressed in healthy donor (HD) CD34 cells, P6 CD34 cells, as well as P6 NK cells. It is not detected (ND) in P6 PMN, CD14, CD3, CD19 cells. The elevated 5′/3′ ratio suggest accumulation of truncated transcript relative to full-length transcript. **c** P6 Clonality at 30 m based on VISA. **d** Single-cell colony assay identified clones with multicopy transgenes. **e** Karyotype in iPSCs derived from P6 peripheral blood CD34+ cells. **f** *HMGA2*-10+ copy clones are the most abundant clones in peripheral CD34+ cells in PT6 and this clone is significantly enriched in iPSC cells derived from CD34+ cells (*p = 0.0002, Fisher’s exact test). Source data are provided as a Source Data file.

There is a previous report of lentivector insertion-mediated activation of *HMGA2* causing a large expansion of a single clone restricted to the erythroid lineage in human lentiviral HSPC β-thalassemia gene therapy trial^6,13^. The activation was caused by alternative splicing of the *HMGA2* transcript to the integrated lentivector LTR which increased the stability of the transcript. To evaluate for potential alternative splicing as a mechanism for the *HMGA2* clonal expansion, our initial assessment by reverse transcription of RNA from patient CD14+ PMN myeloid cells which generally harbored the highest level of HMGA2 VIS failed to detect any endogenous *HMGA2* transcripts. Since *HMGA2* is highly expressed in early embryonic development and is turned off with differentiation in adult tissues except in some stem cells including CD34+ HSC, we reasoned that the high frequency of HMGA2 VIS clones in the short-lived myeloid CD14+ cells may simply reflect an increased pool of *HMGA2* VIS clones in the upstream progenitors from which they are derived. To evaluate the upstream progenitors, we isolated peripheral blood (PB) CD34+ cells from P6 who had a dominant *HMGA2* VIS clone at 18 months following treatment, and by RT-PCR, identified a fusion *HMGA2*-lentivirus transcript (Fig. 3a). Sequencing of the fusion transcript confirmed the alternate splicing mechanism generated by the splicing donor (SD) of *HMGA2* exon 3 and a cryptic splicing acceptor (SA) in the cHS4-400 element in the vector LTR (Supplementary Fig. S1).

We then quantified the expression of the fusion transcript (containing only the first 3 exons) relative to the full-length *HMGA2* transcript (includes exons 4, 5) using the ratio of exon 1–2/exon 4–5. The ratio in healthy donor (HD) CD34^+^ cells was ~1, while in P6 CD34^+^ cells the ratio increased to 5.91 (Fig. 3B), consistent with an accumulation of truncated fusion transcripts of *HMGA2* that lack exon 4–5 and the 3' UTR because of the lentivirus integration. *HMGA2* transcript could also be detected in NK cells from P6 but with an exon 1–2/exon 4–5 ratio of 7.55 (Fig. 3B). Since both the full and the truncated transcripts retained the same endogenous promoter, it was interesting that HMGA2 expression was detected in CD34+ as well as NK cells, suggesting a physiological role for HMGA2 in these cell types, but not in differentiated PMN, CD14+, T or B cells. This expression pattern was also confirmed by RNAseq. The fusion transcript of HMGA2-cHS4 has been detected by RNAseq in P6 CD34+ cells and in CD34+ cells from other patients. These data confirm that the truncated *HMGA2* fusion transcripts initiated in CD34+ cells led to their clonal expansion that was reflected in the short-lived PMN and CD14+ cells that served as useful surrogate biomarkers for the genetic composition in the rarer CD34+ progenitors from which they were derived.

### Expanded HMGA2 clones are commonly associated with multicopy transgenes

In P6, the largest HMGA2 clone remained stable from 24 m to 48 m in CD34+ cells, which constituted about 4% of VIS in PMN, CD14, or CD34 cells. Strikingly, there were ten other VIS tracked at the high frequency throughout this time course, also observed in multiple cell lineages (CD19, NK, PMN, and CD14+ cells) (Figs. 1B, 3C). This synchronized kinetics of multiple VIS was also observed in other patients (Fig. 1B), raising the possibility that HMGA2 insertion and multiple other insertions resided in the same cell clones. To evaluate this possibility, we established single-cell CD34+ colonies from P6 (at 36 months) for vector integration site (VIS) or ddPCR analyses, and found that 20/88 (22.7%) of them harbored the same (10 inserts) or closely similar (11 inserts) with one additional integration, possibly acquired during in vitro transduction, in the *TALDO1* gene (Fig. 3D). Frequencies of specific VIS out of total VIS are commonly used as a surrogate to estimate the frequency of the cell that carry the VIS. However, the presence of multiple inserts per cell give rise to a much larger total number of clones than number of cells. Using the frequency of a VIS alone therefore underestimates the contribution of a multi-insert cell clone and the degree of clonal expansion. To address this, we used ddPCR to quantify all integration site junctions in this set relative to a human genome reference gene and confirmed from multiple assays a true cell clone frequency of 23% of CD34+ and CD14+ cells from P6, much higher than ~4% estimated by VIS (Fig. 3C). We developed a simple statistical method to predict potential multicopy cell clones based on VIS frequency correlation coefficient (Supplementary Methods, Figs. S2 to S4). Based on this approach, we identified numerous other multi-insert clones in many of the patients (Fig. S4 and Supplementary Data 1), that generally also harbored an insert in *HMGA2*, suggesting that while insertion in the *HMGA2* may drive the clonal expansion, the association with multiple copies of transgene also raises the possibility that activated *HMGA2* works in synergy with high dose of IL2Rγc for the observed growth advantage.

### Enrichment of P6 *HMGA2* 10 + copy clone in induced pluripotent cells

Single-cell CD34+ colonies provide limited DNA or RNA, challenging for detailed molecular characterization. To further characterize the P6 HMGA2 10+ copy cell clone and its impact on host gene expression, we established single cell derived induced pluripotent (iPS) stem clones from PB CD34+ cells from P6. Karyotyping showed that these iPS cell clones had a normal karyotype (Fig. 3E). Over half (56%) of the iPSCs (15 of 27 total) harbored the *HMGA2* 10+ clone which is significantly higher compared to CD34+ cells (23%) (*p* = 0.0002, Fisher’s Exact test) (Fig. 3F). This may be attributable to higher expression of HMGA2 and/or IL2RG transgene in the more primitive iPSCs.

### cHS4-400 cause widespread alternative splicing of host genes that it integrates into in the sense (forward) orientation

**Fig. 4:**
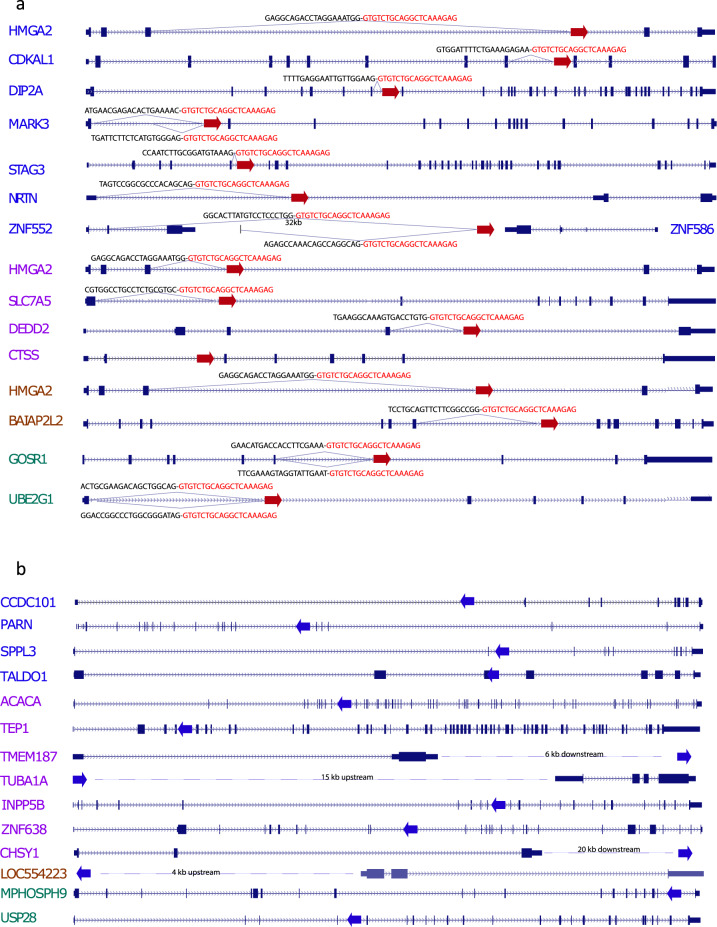
Cryptic splice acceptor in cHS4 causes transcription termination and aberrant fusion transcripts when inserted in same orientation of almost all target genes confirmed in iPS clones. No fusion transcript is observed for proviral integration with reverse orientation as the gene. Four iPSC clones were analyzed by RNAseq. Gene labels with the same color denote LV-insertion in the same iPSC clone. **a** Almost all LV insertions that are in the same orientation as the insert gene generated fusion transcripts of the upstream exons or even upstream introns into cHS4 in the LV, except for CTSS (not expressed in iPSCs, and therefore not possible to detect transcripts). Red arrows denote LV-insertion site in the gene in the same orientation. Fusion transcript sequences were identified by RNAseq and labeled above the junction. Black text indicates the sequences from upstream exons/introns and red text indicates the sequences derived from cHS4. **b** LV insertions in the reverse orientation of target gene or outside the gene do not produce fusion transcripts. Blue arrows indicate LV integration site and orientation relative to the gene. Insertion in *TALDO1* is the only exonic insertion.

To determine if the *HMGA2*-Exon3-cHS4 splicing is sequence context dependent specifically for *HMGA2*, or a more general vector-specific phenomenon, we performed RNA sequencing of nine iPSC clones harboring different sets of integrations, each with multiple copies of VIS in different genes (Supplementary Excel Table 2). The RNAseq analysis supports the latter addition of *TALDO1* insertion to the 10+ clone, instead of a later loss of *TALDO* gene together with the vector insert from the 11+ clone, ascertained by the *TALDO1* expression in #21 #26 (no *TALDO1*) being twice that of #10#11 (with *TALDO1* insertion), consistent with no loss of an allele in #21#26, but that insertion in *TALDO1* exon 3 in clone #10#11 knocked out the expression of one allele. Details of additional iPSC clones are provided in Supplementary Table 2, of the 29 inserts (3 different VIS in *HMGA2*, and 26 in other genes), 14/29 were in introns and in the same orientation as the coding sequence. Chimeric transcripts of target genes fused to the cHS4-400 SA were detected in 93% (13/14) of the same orientation integration events, with only one exception, namely *CTSS* which is not significantly expressed in iPSCs (Fig. 4, Supplementary Data 2). In a few genes such as *MARK3*, *UBE2G1*, and *GOSR1*, multiple fusion transcripts were detected in addition to the expected upstream exons. One VIS did not affect the nearest gene *ZFN586* which is in the antisense orientation. Instead, it resulted in fusion transcripts between cHS4-400 SA of this insertion and another much further away gene *ZFN552*, which is upstream in the sense orientation. The splicing occurred with ZFN552 exon2 SD, skipping ZFN552 exon3, and fused to cHS4-400 SA, >32 kb 3′ to the exon2 SD. Figure 4 summarizes all insertions in each target gene in these clones and illustrates fusion transcripts discovered by RNAseq. Our data show that cHS4 in the vector can cause widespread fusion transcripts with host genes when inserted in the sense (forward) orientation.

### cHS4 is a potent gene trap when integrated in the sense orientation

**Fig. 5:**
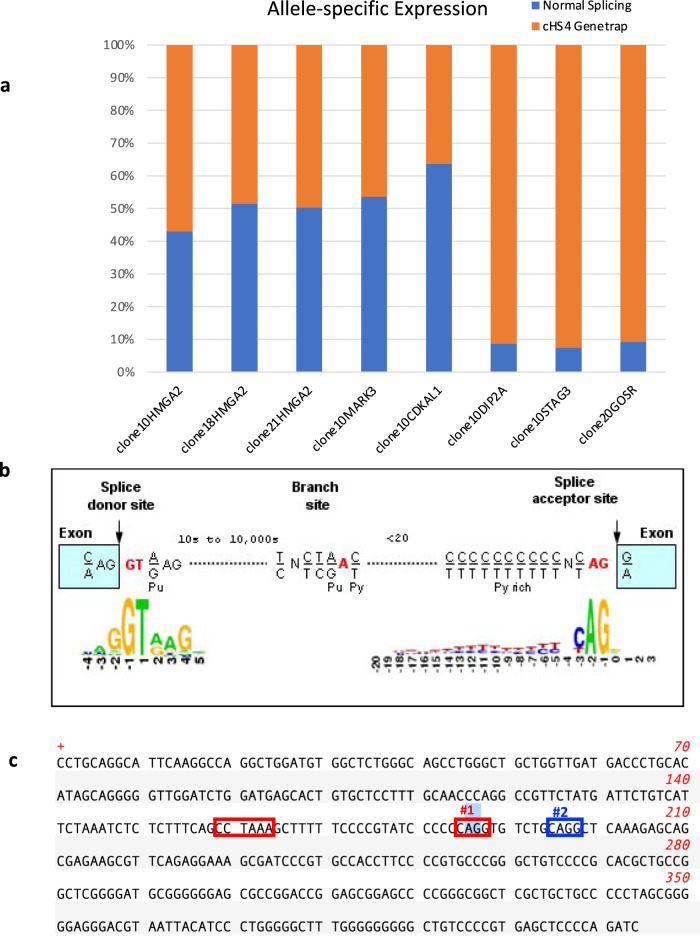
Dominant termination of insert alleles due to a cryptic splice acceptor in the cHS4 region of the vector. **a** Quantification of exon-cHS4 gene trap splicing and normal exon-exon splicing of target genes in single-cell iPS clones. RNAseq was perform for the iPS clones. Read counts across the exon-cHS4 splicing junction and normal exon-exon junction are reported as fraction of the total reads to measure the frequency of each splicing event. **b** Figure depicting the consensus sequences for splice donor, branch and acceptor sites that corresponds to the lentivector cHS4 sequence. **c** Vector sequence indicating branch site and cryptic splice sites. Single base-pair changes from A to T were introduced at #1 and #2 to modify the vector.

We next asked if the cHS4 is a strong gene trap by testing the extent of its impact on the host gene transcription. Low-level alternative splicing of host genes with integrated lentivectors is known to happen^14,15^. We quantified the impact of transcription termination of cHS4 on host genes. By comparing the sequence read numbers of the upstream exon-cHS4 gene-trap event and the normal exon-exon splicing event (Fig. 5a), where insertion causing loss of one allele should result in a 50:50 ratio in RNAseq. Our data demonstrated that for many genes, the fusion transcripts of exon-cHS4 were detected at similar (*HMGA2, MARK3, CDKAL1*) or even higher level (*DIP2A*, *STAG3*, *GOSR*) comparing to normally spliced transcript. For *HMGA2*, the data here seemed to contradict what was observed in patient CD34+ HSC or NK cells. The ratio of the fusion transcript versus the normal transcript was roughly equal in iPS cells, whereas it was much higher in CD34+ or NK cells. This is attributable to the lack of mature let-7 miRNAs in iPSCs and embryonic stem (ES) cells. Mature *let-7* miRNAs level increases significantly in HSCs during development or differentiation of ES cells and vice versa for its negative regulator *LIN28*^16–19^. This is consistent with our RNAseq data showing high expression of *LIN28* in iPSCs and much lower level or absence in CD34+ cells or NK cells. In total, these data show that the cryptic splice acceptor in cHS4-400 is a very strong gene trap that can cause orientation-specific transcription termination of any genes it integrates into via its splicing acceptor and polyadenylation signal in the LTR. It can even cause dysregulation of target genes even if it is integrated outside the gene. This could lead to either gain of function if the truncated protein has new functions or loss of function of the target genes that are needed for cellular growth.

### Clonal expansion of cells with vector insertion in other genes

Next, we evaluated for potential gene trapping in other genes to account for their expansion, which could also have potential implications. A notable clone was one identified in P3 at 20 months following transplant that accounted for ~50% of his NK cells and harbored only one VIS in the sense orientation in *NF1*, a tumor suppressor gene, the gene product of which negatively regulates Ras activity. Heterozygous *NF1* mutations lead to neurofibromatosis type 1 and in a variety of tumors, including juvenile myelomonocytic leukemia^20,21^. Anxieties regarding the clone contributing to ~50% of the patient’s NK cells were balanced by the lower-than-normal numbers of peripheral blood NK cells, and by his stable clinical status. This *NF1*+ clone was stably maintained at high frequency through the last assayed time point at 54 months, with his absolute NK cell count still below normal range. RNAseq analysis on three NK cell RNA samples from P3 revealed a fusion transcript of *NF1* upstream exon to cHS4 in two out of three samples that was subsequently also identified in other cell types, though to a lesser degree. Also of note was a VIS in the *ETS1* oncogene in CD3+ cells in P6 (10% of VIS) that also remained stable from 36–48 months. These examples highlight the expanse of genes that may be impacted by the cHS4 gene trap activity and lead to clonal expansion. However, it is not easy to establish the causal relationship in each case.

### Site-directed mutation of the SA in cHS4 eliminates gene trap activity

**Fig. 6:**
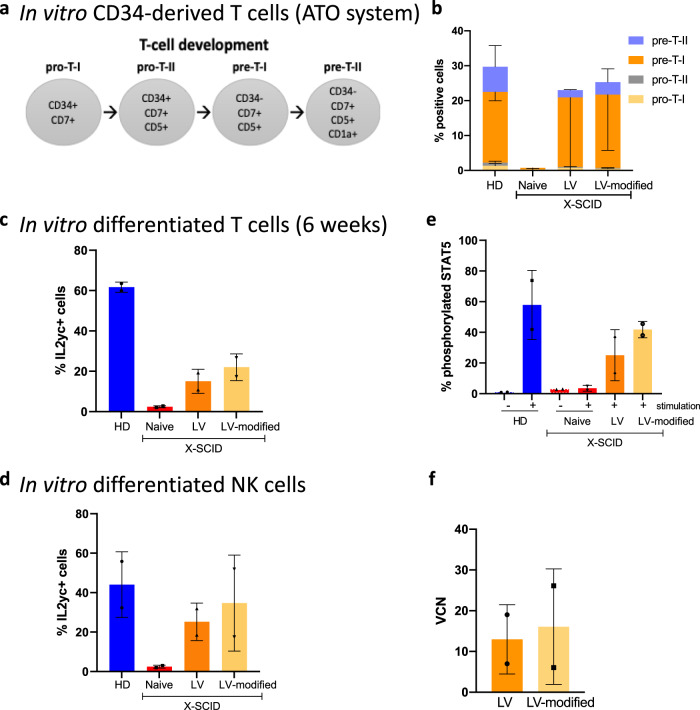
Modified vector with removal of cryptic SA corrects X-SCID CD34+ HSCs. Functional restoration of X-SCID CD34+ HSCs transduced by modified lentivector. **a** Stages of T-cell differentiation using the Artificial thymic organoid. **b** Percentages of in vitro CD34-derived T-cell progenitors. **c** Percentages of γ+ cells (by flow cytometry) following in vitro CD34+ differentiation into T cells. **d** Percentages of γ+ NK cells (by flow cytometry) following in vitro differentiation. **e** Phospho-STAT5 signaling in in vitro-differentiated T cells derived from transduced X-SCID CD34+ HSCs. **f** Vector copy number determined by ddPCR in HSCs transduced by clinical lentivector (LV) or the modified vector (LV-modified). For all graphs, data are showed as mean ± standard deviation (SD); *n* = 2 independent experiments. Source data are provided as a Source Data file.

Next, we removed the cryptic SA in the cHS4 region of the vector by site-directed mutagenesis, located outside the CTCF binding site in order to have minimal impact on the insulator function. The conserved AG dinucleotide sequence for SA (Fig. 5B, C) was changed to a TG dinucleotide in the modified vector. We transduced SCID-X1 patient CD34+ HSPCs with either the clinical Cl20-i4-hγcOPT vector or the modified version. We performed in vitro IL2Rγc-dependent differentiation of transduced HSCs into T cells using an artificial thymic organoid system^22^ (Fig. 6a). SCID-X1 patient CD34+ cells transduced with modified vector was analyzed by flow cytometry (analysis schema shown in Supplementary Fig. S5) maintained T-cell differentiation properties (Fig. 6B), IL2RGγc expression in in vitro-differentiated T cells (Fig. 6C) and NK cells (Fig. 6D), as well as restored IL2RGγc-STAT5 signaling (Fig. 6E), comparable to the original clinical vector, confirming that the minor change to the cryptic SA did not disrupt vector function or transducing capability (Fig. 6F). In vitro assessment confirmed IL2RGγc expression in transduced patient cells that indicates the modification did not disrupt transgene function. To investigate whether the gene trapping activity is removed from the new vector, we performed RNAseq on CD34+ colonies as either single colonies (to increased sensitivity) or pooled colonies (to increase the chance of detecting various trapped genes). No fusion transcripts indicative of gene trapping activity were identified in cells transduced with the modified vector, while many fusion transcripts were identified in cells transduced with the clinical vector, indicating successful functional removal of the described SA.

## Discussion

Although no cancers were reported with over a hundred patients treated with lentiviral gene therapy over the past 12–15 years, the recent *MECOM*-related leukemia in one patient with adrenoleukodystrophy treated with a SIN-lentivector (bluebird bio) reaffirms that insertional mutagenesis remains a safety concern for gene therapy using integrating vectors. Long-term surveillance remains critical as events may present late, as demonstrated in the γRV-treated SCID-X1 patient and adenosine-deaminase deficient SCID patient many years after treatment^23^. Clonal expansion of *HMGA2* VIS clones was noted in our first two patients of SCID-X1 gene therapy following a relative short time period. Long-term surveillance of these clones revealed the kinetics of these clones that were also identified in the entire cohort of treated 8 patients, indicating a system-wide problem related to the cryptic SA in the cHS4 region of the vector. Single-cell clone derived RNA sequencing data characterized fusion transcripts of multiple genes, including tumor suppressor genes.

Integrating vectors may trigger oncogenesis by (a) interactions between viral enhancer sequences and cellular promoters to cause transcriptional activation and dysregulation of oncogenes^24–26^, (b) insertion of vector splicing and polyadenylation signals that may lead to dysregulation of gene expression through aberrant splicing, premature transcript termination, and the generation of chimeric transcripts. The recent SIN lentivirus-related leukemia in the ALD patient raises another mechanism through a strong internal promoter (MND) that upregulated a nearby oncogene (Bluebird Bio). Our report highlights risks associated with the preferential integration of lentivectors within transcription bodies that also increases risks of aberrant transcripts. Although aberrant transcripts have been demonstrated in vitro^14,27–29^ and in a tumor model^30^, the long-term biological impact in humans following lentivector gene therapy remains unclear.

Analysis of the gene-trapping events revealed interesting biology in vivo not previously appreciated. The *HMGA2* gene encodes a small protein with three DNA-binding AT-hook domains that modulates chromatin structure, transcription, and epigenetic regulation of multiple genes^31,32^. It plays crucial roles in the proliferation, cell-cycle progression, apoptosis, and senescence of cells, and given its high expression level selectively in primitive embryonic tissues, it is not surprising that *HMGA2* has been recently shown to be involved in self renewal of hematopoietic stem cells^33,34^ and neural stem cells^35,36^. *HMGA2* clonal expansion in our patients was first noticed in peripheral blood, especially myeloid cells. However, *HMGA2* transcripts were not identified from circulating myeloid cells, but only from CD34+ cells, highlighting the importance of surveilling expressing cells. While PMNs with short half-life are a good surrogate for stem and progenitor cells with regards to genomic VIS, the transcriptional profile is highly dependent on the specific cell stage. Our data demonstrated that lentiviral insertion-mediated activation of *HMGA2* can provide a growth advantage for CD34+ cells and supports its role in hematopoietic stem cell renewal and promoting survival and proliferation after stem cell transplantation.

The activation of *HMGA2* in our patients caused by the cHS4 insulator element in the lentivector was also observed in β-thalassemia gene therapy that used a similar cHS4 element containing vector where the same SA was involved in the alternative splicing^6^. In contrast, *HMGA2* clonal expansion has not been reported in lentivector gene therapies for other rare single-gene diseases such as Wiskott-Aldrich syndrome^4^, metachromatic leukodystrophy^5^ or chronic granulomatous disease^2^ using vectors without the cHS4 insulator, thereby indicating that the gene trapping activity is vector specific. Interestingly, although the expanded *HMGA2* VIS clone was restricted to the myeloid lineage in the β-thalassemia study, we identified *HMGA2* VIS clones in both myeloid and lymphoid lineages in our study, raising the possibility that there may be additional factors affecting the clonal expansion of *HMGA2* clones. A key difference between our studies is the transgene itself, where the multiple *IL2RG* transgenes in the present study may super-impose an additional growth advantage. Interestingly, Wang et al. reported enrichment of *HMGA2* clones in two SCID-X1 patients treated with γ retroviral gene therapy^37^ even though the vector did not involve cHS4, supporting the concept that the *IL2RG* transgene may play a synergistic role in *HMGA2* clonal expansion.

The chromatin insulator was introduced to act as a barrier to prevent silencing of the transgene or interference with the host cell or transgene expression as an enhancer blocker. Two copies of insulators flanking the transgene are deemed necessary to achieve protection from gene silencing and enhancer blocking. Since the large (1200 bp full length cHS4) size and repetitive nature can generate problems with instability in the lentivector LTR and reduce the titer of the virus, a shorter 400 bp fragment with full protection function was used instead. Our data suggest that cHS4 should not be used in its current form due to its strong transcription termination activity. There are many other cryptic splicing sites in lentivectors, although most of them are known to cause only low-level alternative splicing of host genes^14^. We did not identify any other aberrant transcripts in this study, although it is possible that less active cryptic splice sites may manifest in the absence of cHS4 SA. We demonstrated that introduction of a simple point mutation in the cHS4 SA eliminated its gene trap activity and recommend such modification for clinical lentivector containing splice elements to reduce genotoxicity risks.

While the proliferative advantage from increased *HMGA2* expression in CD34+ cells may appear advantageous for a CD34+ transduced cell product, its potential causative involvement in multiple types of cancers^31,38^ poses a safety concern that requires long-term follow up. The concerns are somewhat offset by our observations that to date, (1) our vector has not caused uncontrolled expansion, and (2) there has been spontaneous attrition of the *HMGA2* VIS clones after a peak at 1–2 years post treatment in most patients. We attribute the limitation of *HMGA2* effects from increased stability to regulation by its endogenous promoter restricting its expression to specific phases and lineages of cell development. Increased levels of the truncated *HMGA2* protein may provide an advantage to CD34+ HSCs and animal models to evaluate whether the potential benefits and safety issue with *HMGA2* dysregulation for hematopoiesis would be of value. In a study with a transgenic mouse model that expressed 3′UTR truncated *HMGA2*, a growth advantage was seen for *HMGA2* expressing bone-marrow cells after serial transplantation, but no malignancy was observed^39^. In a recent rhesus macaque model^40^ transplanted with CD34+ cells transduced with a lentivector to constitutively over-express 3′ UTR truncated *HMGA2* under control of the MSCV promoter, these cells demonstrated increased self-renewal potential that was self-limiting and did not result in any hematological malignancies even after three generations of transplantation^40^.

An alternative mechanism likely accounts for the expansion of the *NF1* VIS clone in P3 NK cells if it is not a stochastic event. The strong cHS4 gene trap activity can have two different consequences, the commonest being a loss of function for one allele of an autosomal gene, that can be compensated by the other functional allele. However, loss of one allele may lead to haplo-insufficiency that may be problematic for tumor suppressor genes such as *NF1*, a potential cause of dysregulated hematopoiesis^41,42^. Alternatively, the cHS4 gene trap may result in truncated transcripts/proteins with a gain of function as demonstrated by the increased stability of *HMGA2* fusion transcript. Most of the truncated HMGA2 transcripts retain the first three exons, which encode all three AT-hook DNA-binding domains of the protein. The truncated protein is functional in providing a growth advantage to cells in which it is expressed. Cesana et al. and colleagues showed that the truncated protein, even though lacking in the C-terminal domains, is highly comparable to the full-length protein in chromatin binding patterns and overexpression of either the short or long HMGA2 protein can promote engraftment of conditionally immortalized iPSC-derived hematopoietic progenitors and human bone-marrow HSPCs in mouse^19^. Our data showing enrichment for multi-insert clones suggest that the multiple copies of *IL2RG* may augment *HMGA2* activation, acting in synergy to provide a growth advantage.

In summary, we have reported potent gene trapping due to a cryptic splice acceptor in the lentivector cHS4 region of the *IL2RG* vector causing substantial increase in *HMGA2* clones in CD34+ cells. Identification of the aberrant fusion transcripts is dependent on the use of appropriate cells in which the genes are expressed. A simple mutagenesis of the strong SA removed the gene trapping activity to improve safety of the vector.

## Methods

### Human subjects

This is a phase 1/2 nonrandomized clinical trial of ex vivo hematopoietic stem and progenitor cell (HSPC) gene transfer treatment for SCID-X1 using a SIN insulated lentivirus. The study is approved by the National Institute of Allergy and Infectious Diseases (NIAID) Institutional Regulatory Board (clinical protocol #11-I-0007, ClinicalTrials.gov ID NCT01306019) and Institutional Bio-safety Committee, FDA (investigational new drug #15041), sponsored by the NIAID Regulatory Compliance and Human Subjects Protection Branch.

We designed this study to be a non-randomized clinical trial of gene transfer using a self-inactivating, insulated, lentiviral gene transfer vector to treat patients with X-linked severe combined immunodeficiency (SCID-X1) who are between 2 and 30 years of age; who do not have a tissue matched sibling who can donate bone marrow for a transplant; who may have failed to obtain sufficient benefit from a previous half-tissue matched bone-marrow transplant; and who have clinically significant impairment of immunity (Table 1). Mutations and clinical status prior to treatment for the first five patients were previously reported^3^.

Primary outcomes were level of transduction and immune reconstitution. The primary outcome of immune reconstitution efficacy is determined by laboratory assays such as vector copy number (VCN) and clinical assessments such as T- and B-cell reconstitution (Table 1).

Secondary outcomes were rates of serious adverse events and clonal expansion. The secondary endpoints are to determine serious side effects related to gene transfer and surveillance of vector integration sites. Limited early vector insertion data was previously reported^3^.

The inclusion and exclusion criteria for participation in the study are the following: a proven IL2RG mutation, completed HLA typing, no available HLA matched sibling donor, between 2 and 40 years of age and weigh >10 kg. If previously transplanted, patient must be >18 months post-transplant, additional inclusion criteria are expected survival of >120 days, proven HIV negative status, have suitable family and social circumstances able to comply with protocol procedures and long-term follow up requirements, documented B cell dysfunction or dependent on gamma globulin supplementation, willingness to store blood and tissue samples, and satisfy laboratory (lymphopenia, lymphocyte dysfunction) and clinical criteria of immune dysfunction and significant clinical disease such as infections, immune dysregulation, chronic lung disease, gastrointestinal disease, malnutrition, failure to thrive, skin problems (molluscum, warts, mucocutaneous candidiasis). Exclusion to participation are subjects with hematologic malignancies, treatment with chemotherapy, HIV1 infection, and hepatitis B infection.

### Lentiviral vector

The Cl20-i4-EF1α-hγcOPT used in this clinical trial is a vesicular stomatitis virus G (VSV-G)–pseudotyped, third-generation SIN vector that uses a promoter fragment from the eukaryotic EF1α gene to express a codon-optimized human γc cDNA and contains a 400-bp cHS4 insulator fragment from the chicken β-globin locus within the SIN LTR (*1, 3*).

### Autologous CD34^+^ HSPC collection and isolation

Patients received G-CSF at 16 mg/kg per day by subcutaneous injection for five consecutive days, supplemented by plerixafor (0.24 mg/kg, sc, 11 h before collection) [National Institutes of Health (NIH) protocol 94-I-0073] before apheresis at the NIH Clinical Center. The products were processed using FDA-approved Isolex immune anti-CD34 magnetic bead system to isolate and enrich CD34^+^ cells by the NIH Department of Transfusion Medicine Cell Processing Facility. Purified CD34^+^ HSPCs were cryopreserved until gene therapy.

### Transduction of CD34^+^ HSPCs

Ex vivo culture and transduction of the patient’s autologous CD34^+^ HSPCs with VSV-G–pseudotyped Cl20-i4-EF1α-hγcOPT LV were performed and certified by the NIH Department of Transfusion Medicine Cell Processing Facility. Transduction involved thawing and suspension of patient CD34^+^ HSPCs in X-VIVO 10 serum-free growth medium [containing 1% human serum albumin plus cytokines (stem cell factor, 50–100 ng/ml; Flt-3 ligand, 50–100 ng/ml; thrombopoietin, 50–100 ng/ml; IL-3, 5 ng/ml)]. The cells were cultured in T-175 tissue culture flasks coated with the recombinant fibronectin fragment (RetroNectin) and exposed to lentivirus for 6 to 8 h each day for two consecutive days after an overnight pre-stimulation. Transduced CD34^+^ HSPCs were harvested and infused fresh after required safety testing and quality control testing.

### Conditioning regimen

A 6 mg/kg busulfan dose given over 2 days was used prior to infusion of transduced cells. No T cell–or B cell–depleting agents were given.

### Clinical course

No adverse events were noted with the infusion of the transduced CD34^+^ HSPC product. The nadir for the expected busulfan-related effects of neutropenia and thrombocytopenia occurred at 2–3 weeks after treatment followed by recovery without requiring any cellular support or intervention. Subjects were discharged home by 3–4 weeks after gene therapy. Three of the eight patients developed febrile neutropenia that responded to empiric antimicrobial therapy.

### Cell lineage separation for gene marking and integration analysis

After polymorphonuclear (PMN) cell separation using histopaque, the mononuclear leukocyte layer was fractionated by magnetic beads per manufacturer’s instructions (Dynal Beads, Invitrogen).

### Peripheral blood CD34^+^ cell analysis for VCN and insert site analyses

PBMCs were purified from non-mobilized peripheral blood by Ficoll separation (Lymphocyte Separation Medium, MP Biomedicals). CD34^+^ cells were isolated from PBMCs using magnetic cell sorter magnetic beads (Miltenyi Biotec) according to the manufacturer’s protocol. CD34^+^ cells were expanded for 8–12 days in StemSpan II media (STEMCELL Technologies) supplemented with 100 ng/ml each of human stem cell factor, FLT-3L, and thrombopoietin (PeproTech). DNA was isolated using the DNeasy Blood and Tissue Kit (QIAGEN).

### Quantitative determination of VCN by ddPCR

To measure the vector-carrying cells, we used a ddPCR (Bio-Rad) assay. The ddPCR assay allows the measurement of absolute copy number without using standard curve. The vector-specific primers and probes are as follows: HIV forward, 5′-CTGTTGTGTGACTCTGGTAACT-3′; HIV reverse, 5′-TTCGCTTTCAAGTCCCTGTT-3′; and HIV probe, 5′-/56-FAM/AAATCTCTA/ZEN/GCAGTGGCGCCCG/3IABkFQ/-3′. We multiplexed a reference gene assay for cell counts in the same reaction [myocardin-like protein 2 (MKL2): forward, 5′-AGATCAGAAGGGTGAGAAGAATG-3′; reverse, 5′-GGATGGTCTGGTAGTTGTAGTG-3′; and probe, 5′-/56-HEX/TGTTCCTGC/ZEN/AACTGCAGATCCTGA/3IABkFQ/-3′]. Cell number was calculated as half of the *MKL2* counts because each cell is diploid. VCN was calculated as vectors per cell.

### Quantification of clonal expansion of specific integration site

Top expanded clones identified by the VISA sequencing assay were followed up and monitored by specific ddPCR assay. All of our ddPCR assays consisted of a common LTR primer, an LTR probe, and a specific primer for genomic DNA junction (5LTR reverse, 5′-CTGCAGGGATCTTGTCTTCTT-3′; 5LTR junction probe, 5′-/56-FAM/TGGAAGGGC/ZEN/TAATTCACTCCCA/3IABkFQ/-3′; 3LTR forward, 5′-CCCACTGCTTAAGCCTCAATA-3′; 3LTR junction probe, 5′-/56-FAM/AAGTAGTGT/ZEN/GTGCCCGTCTGTTGT/3IABkFQ/-3′). We multiplexed the integration site-specific assay together with the *MKL2* reference gene assay, which measured cell counts in the same reaction.

### Vector Integration Site Analysis (VISA)

Patient DNA was subjected to VISA as described previously (3).

### CD34^+^ single cell clone VISA assay

To verify multi-copy transgene integrated in the same cell, we isolated CD34^+^ cells from patients and performed colony-forming cell (CFC) assays in methylcellulose media. Colonies formed from single cells were plucked and transferred into 50 µL of water and then Proteinase K was added for digestion. Lysate (9 µL) containing cellular DNA was used for whole genome amplification using GenomePlex Single Cell Whole Genome Amplification kit WGA4 (MilliporeSigma). The amplified DNA was subjected to VISA and ddPCR analysis. Integration sites identified from single colonies are considered from single cell.

### iPSC single cell clones

We established iPSC single cell-derived clones from patient CD34^+^ cells by Sendai virus reprogramming as previously described *(32)*. The established single-cell clones were subjected to VISA and ddPCR analysis. G-band karyotype analysis was performed commercially (WiCell).

### Detection of HMGA2 fusion transcripts

Total RNA was extracted from patient or healthy donor blood cells. Reverse transcription was carried out to generated cDNA using Invitrogen kit. PCR was performed using nested primers, with forward primers matching *HMGA2* exon 2 and exon 3, and reverse primers matching the R region of lentivector LTR (HMGA2-Ex2-F CGGTGAGCCCTCTCCTAA; Vector-HIV-R AGCAGTGGGTTCCCTAGTTA; HMGA2-E3-M13Fnest GTAAAACGACGGCCAGTGCAGAAGCCACTGGAGAAA; Vector-HIV-R-M13Rnest GGAAACAGCTATGACCATGCCAGGCTCAGATCTGGTCTAAC). Two amplicons were observed with slightly different sizes on the gel. The amplicons of the fusion transcripts were sequenced by Illumina MiSeq. We were able to deduce two different alternatively spliced variants of *HMGA2* exon 3 into the cHS4 insulator core element of the vector.

### Quantification of HMGA2 transcripts

Full-length *HMGA2* and truncated *HMGA2* transcripts were quantified by ddPCR after converting RNA to cDNA by reverse transcription. Two ddPCR assays were designed for HMGA2, one 5′ to the integration site cluster at exon 2/exon 3 junction, the other 3′ to the integration site cluster at exon 4/exon 5 junction. These two assays were used to examine the gene expression of *HMGA2* and the 5′/3′ ratio for the 3′-truncated form that might be caused by vector integration. CD34^+^ cells from healthy donors were used as control. Primer and probe sequences for 5′ assay are forward CCC TCT CCT AAG AGA CCC A, Probe /56-FAM/TT TGC TGC C/ZEN/T TTG GGT CTT CCC /3IABkFQ/, Reverse CTG CCT CTT GGC CGT TT. Primer and probe sequences for 3′ assay are: forward GTT CAG AAG AAG CCT GCT CA, Probe /56-FAM/AG AGT CTG C/ZEN/C GAA GAG GAC TAG GG/3IABkFQ/, Reverse TGC TGA GGT AGA AAT CGA ACG.

### RNAseq

Total RNA was isolated from iPS cell clones. We performed both mRNAseq and total RNAseq using NEB Next Ultra RNA Library Prep Kit for Illumina (E7530S) following to manufacturer’s recommended protocols (New England Biolabs, Ipswich, MA). The libraries were sequenced with 2 × 75 bp paired end reads on Illumina NextSeq2000 with P2 reagents (Illumina, San Diego, CA). Data analysis was performed with NextSeq2000 onboard Dragon RNAseq pipeline. For fusion transcript detection with cHS4, fastq files were used to search all reads that contain the cHS4 SA site. The junctions were then split and sorted and mapped to human genome hg38 to find the fusion partners.

### T cell differentiation: Artificial Thymic Organoid (ATO) system

CD34^+^ HSPCs were differentiated into T cells in vitro using a 3D artificial thymic organoid system (15, 33). Briefly, CD34^+^ cells were co-cultured with MS5 murine stromal cell line modified to express DLL4 ligand in a volume of 5 μL on 0.4 μm transwell inserts (EMD Millipore). Culture medium containing 5 ng/ml rhFlt3-ligand and 5 ng/ml rhIL-7 (PeproTech) was changed every 3–4 days. After 6 weeks, ATOs were disaggregated and filtered through a 70 μm cell strainer before staining for flow cytometry analysis.

### NK differentiation

HSPCs were differentiated into NK cells in vitro using a protocol adapted CD34^+^ cells were resuspended in Iscove’s Modified Dulbecco’s Medium supplemented with 20% Fetal Bovine Serum (FBS) and 50 μM ß-mercaptoethanol containing 5 ng/mL rhIL-3 (first week only), 10 ng/mL rhIL-15, 20 ng/mL rhIL-7, 20 ng/mL rhSCF, 10 ng/mL rhFlt3-ligand and 100 U/mL rhIL-2 for 35 days. 40Gy-irradiated K562-mb15-41BBL cells (kindly provided by St Jude Children’s Research Hospital, Memphis, TN)^43^ at days 7 and 21 of differentiation. Cells were harvested at day 35 for molecular and phenotypic analysis, and cytotoxicity *(34)*.

### Flow cytometry

Flow cytometric analysis was performed using a BD Canto flow cytometer, DIVA software (BD Biosciences) and FlowJo 10.6.1 software (Tree Star) using monoclonal antibodies or apoptosis as listed.

### Statistics

Chi-square test or Fisher’s exact tests were used as described in the text. For prediction of multicopy integration sites in the same cell clone, we calculated the frequency of all integration sites at each timepoint and in each cell lineages. We then calculated correlation coefficient of VIS frequencies. The correlation coefficient and total counts of each VIS is considered together to make calls for multicopy clones.

### Study approval

The study was approved by the National Institute of Allergy and Infectious Diseases (NIAID) Institutional Regulatory Board (clinical protocol #11-I-0007, ClinicalTrials.gov ID NCT01306019) and Institutional Biosafety Committee, FDA (investigational new drug #15041), sponsored by the NIAID Regulatory Compliance and Human Subjects Protection Branch. Written informed consent was received from participants prior to inclusion in the study.

### Reporting summary

Further information on research design is available in the Nature Research Reporting Summary linked to this article.

## Supplementary information

Supplementary Information

Description of Additional Supplementary Files

Supplementary Dataset 1

Supplementary Dataset 2

Reporting Summary

## Data Availability

All source data are provided in Source Data Files for this paper. All patient vector copy numbers and integration site source data in Excel tables are provided in Source Data files. RNAseq data for iPSC clones and P6 patient blood samples are available at NCBI Sequence Read Archive (PRJNA788948: SRR17238208- SRR17238226, which can all be found on the National Center for Biotechnology Information database). Source data are provided with this paper.

## Source data

Source data
