# Supplementary material for: Lentivector cryptic splicing mediates increase in CD34+ clones expressing truncated HMGA2 in human X-linked severe combined immunodeficiency
Source: Nat Commun. 2022 Jun 28;13:3710. doi: 10.1038/s41467-022-31344-x (PMC9240040; doi:10.1038/s41467-022-31344-x)
Supplement: Supplementary file 1 — Supplementary Information [file 41467_2022_31344_MOESM1_ESM.pdf]

Supplementary Materials for

Lentivector cryptic splicing mediates increase in CD34+ clones expressing truncated *HMGA2* in human SCID-X1

Authors:

*Suk See De Ravin[1], Siyuan Liu[2], Colin L. Sweeney[1], Julie Brault[1], Narda Whiting-Theobald[1], Michelle Ma[1], Taylor Liu[1], Uimook Choi[1], Janet Lee[1], Sandra Anaya O'Brien[1], Priscilla Quackenbush[1], Tyra Estwick[1], Anita Karra[1], Ethan Docking[1], Nana Kwatemaa[1], Shuang Guo[2], Ling Su[2], Zhonghe Sun[2], Sheng Zhou[3], Jennifer Puck[4], Morton J. Cowan[4], Luigi D. Notarangelo[1], Elizabeth Kang[1], *Harry L. Malech[1], *Xiaolin Wu[1]

Affiliations:

[1] Laboratory of Clinical Immunology and Microbiology, NIAID, NIH, Bethesda, MD 20892

[2]Cancer Research Technology Program, Leidos Biomedical Research, Inc., Frederick National Laboratory for Cancer Research, Frederick, MD, 21702

[3] Experimental Cell Therapeutics Lab, St. Jude Children's Research Hospital, Memphis, TN, 38105

[4] Division of Allergy Immunology and Blood and Marrow Transplantation, Department of Pediatrics, University of California San Francisco and UCSF Benioff Children's Hospital, San Francisco CA, 94143

* Correspondence:

1. Suk See DeRavin, MD, PhD

sderavin@niaid.nih.gov

2. Harry L. Malech, MD

hmalech@niaid.nih.gov

3. Xiaolin Wu PhD

forestwu@mail.nih.gov

Supplementary information includes:

1. Supplementary Methods detailing the calculations for clonal abundance.
2. Supplementary Figure S1 shows the sequence of the fusion transcript showing the alternate splicing generated by the splicing donor (SD) of HMGA2 exon 3 and a cryptic splicing acceptor (SA) in the cHS4-400 element in the vector LTR.
3. Supplementary Figure S2 shows the dynamics of clonal composition in each cell lineage after gene therapy for P1, P3, P4, P5, P6, P7 and P8. Each color denotes a unique vector insertion clone. The size of each color-coded bar reflects the relative contribution of each insert clone.
4. Supplementary Figure S3 Graphs summarizing the top three vector insertion sites in peripheral blood polymorphonuclear, CD14+ myeloid, and NK cells in P3. The gray, orange and blue graphs represent relative frequencies of vector inserts in *HMGA2*, *SIK3* and *NF1*.
5. Supplementary Figure S4 Graphs summarizing the top seven vector insertion sites in peripheral blood polymorphonuclear and NK cells in P4, color-coded as indicated. The identical kinetics of the multiple inserts, for example, of *HMGA2, DNM2*, *PCNT*, *SCA1, SAE1m IG*FBP2 suggests multiple inserts in a clone, and another clone with *HMGA2* and *KIFC1* and their course following gene therapy.
6. Supplementary Figure S6 Flow cytometric gating strategy for evaluation of *in vitro* T-cell development from CD34+ cells using Artificial Thymic Organoids.
7. Supplementary Table S1 shows top vector integration sites and multicopy clones identified in P1, P3, P4 and P6 by VIS frequency correlation. Detailed information is also provided as Supplemental Data in excel document.
8. Supplementary Table S2 shows fusion Transcripts created by vector gene trapping of targeted genes in inducible pluripotent stem clones. VIS that are in the same orientation of gene and inside introns are highlighted and expected to result in premature termination. Detailed information is provided in Supplemental Data (Excel document).

Figure S1

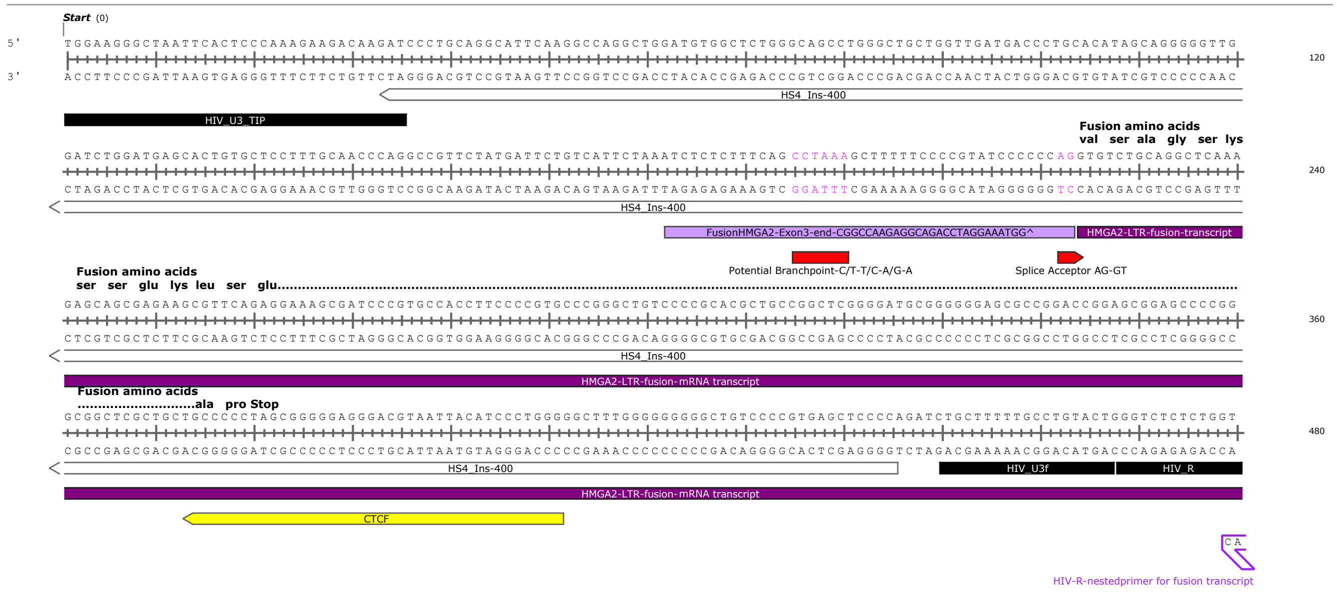

Figure S1

LV CL20-i4-EF1a-hgcOPT insert with HMGA2 splice fusion annotation (Linear, 4638basepairs). Shown is the vector insert sequence following lentivector insertion in the CD34+ cell genome. The genomic sequence of the insert site begins before the shown 5' end and after the 3' end of the vector insert sequence. The figure is shown with annotations as for an insertion into HMGA2 intron 3, and the generation of a truncated fusion RNA transcript when the vector is inserted in the sense (forward) orientation within an intron of any expressed gene.

Figure S2

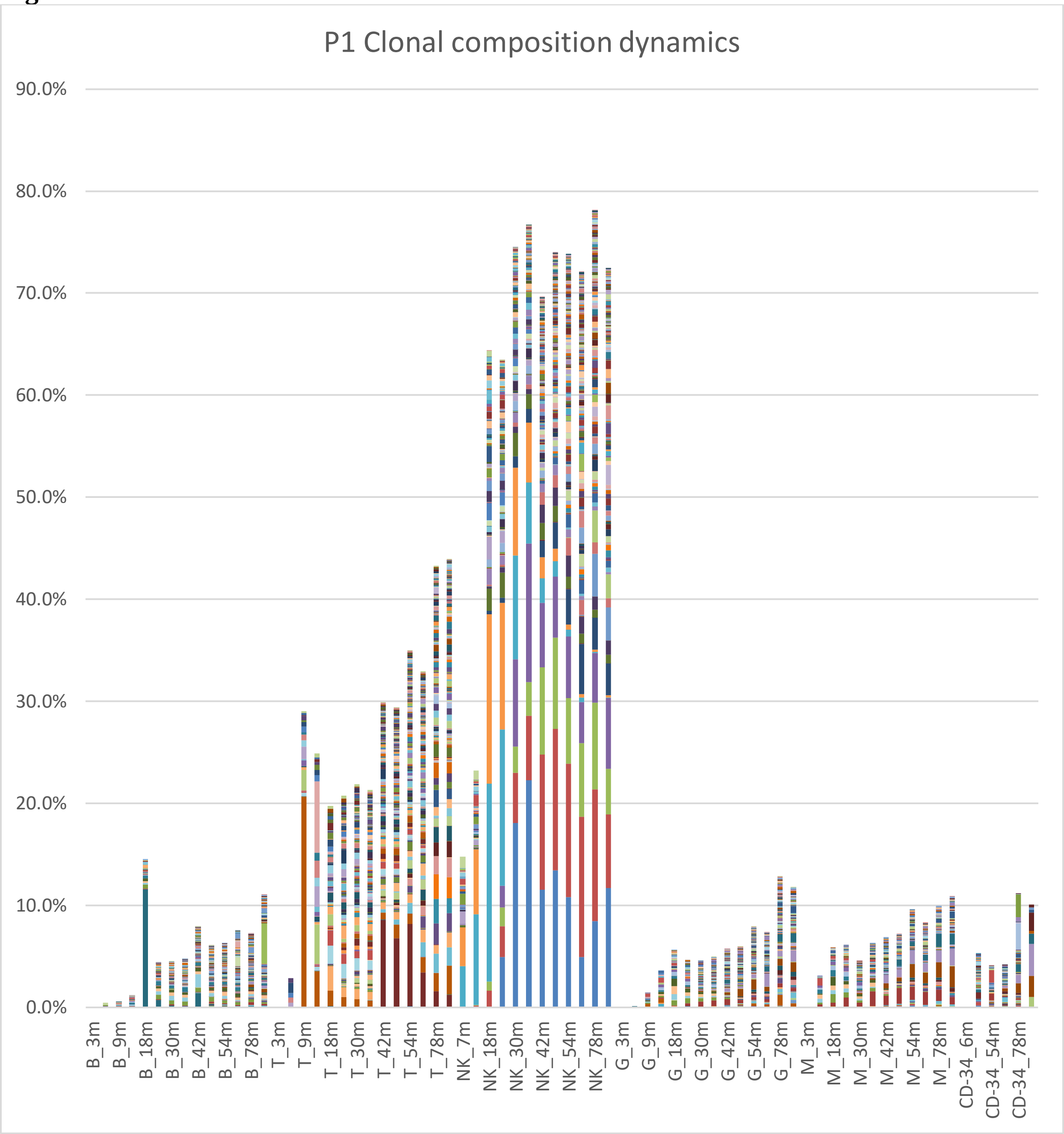

P1 Clonal composition dynamics
90.0%
80.0%
70.0%
60.0%
50.0%
40.0%
30.0%
20.0%
10.0%
0.0%
B_3m
B_9m
B_18m
B_30m
B_42m
B_54m
B_78m
T_3m
T_9m
T_18m
T_30m
T_42m
T_54m
T_78m
NK_7m
NK_18m
NK_30m
NK_42m
NK_54m
NK_78m
G_3m
G_9m
G_18m
G_30m
G_42m
G_54m
G_78m
M_3m
M_18m
M_30m
M_42m
M_54m
M_78m
CD-34_6m
CD-34_54m
CD-34_78m

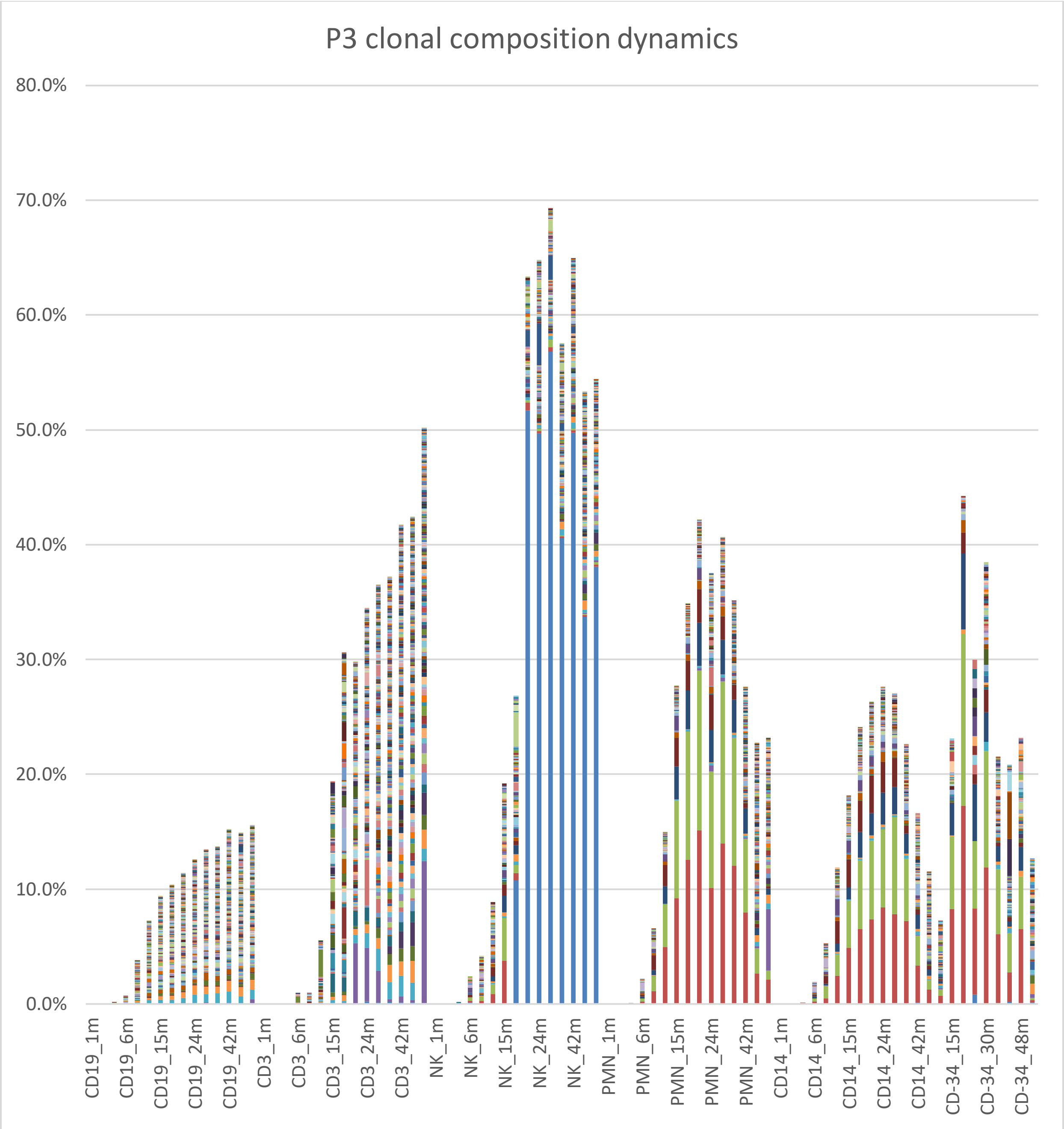
P3 clonal composition dynamics
80.0%
70.0%
60.0%
50.0%
40.0%
30.0%
20.0%
10.0%
0.0%
CD19_1m
CD19_6m
CD19_15m
CD19_24m
CD19_42m
CD3_1m
CD3_6m
CD3_15m
CD3_24m
CD3_42m
NK_1m
NK_6m
NK_15m
NK_24m
NK_42m
PMN_1m
PMN_6m
PMN_15m
PMN_24m
PMN_42m
CD14_1m
CD14_6m
CD14_15m
CD14_24m
CD14_42m
CD-34_15m
CD-34_30m
CD-34_48m

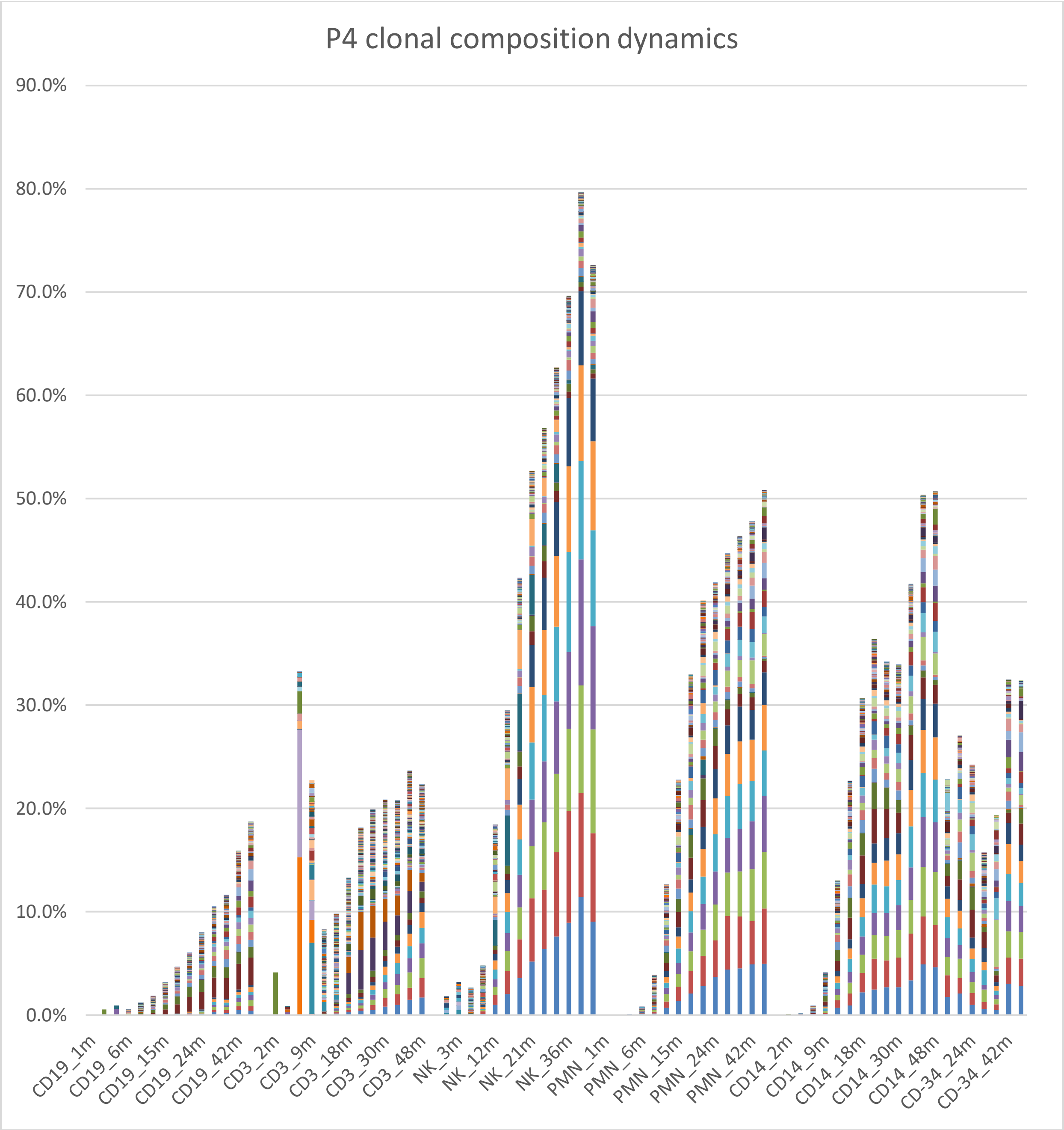
P4 clonal composition dynamics
90.0%
80.0%
70.0%
60.0%
50.0%
40.0%
30.0%
20.0%
10.0%
0.0%
CD19_1m
CD19_6m
CD19_15m
CD19_24m
CD19_42m
CD3_2m
CD3_9m
CD3_18m
CD3_30m
CD3_48m
NK_3m
NK_12m
NK_21m
NK_36m
PMN_1m
PMN_6m
PMN_15m
PMN_24m
PMN_42m
CD14_2m
CD14_9m
CD14_18m
CD14_30m
CD14_48m
CD-34_24m
CD-34_42m

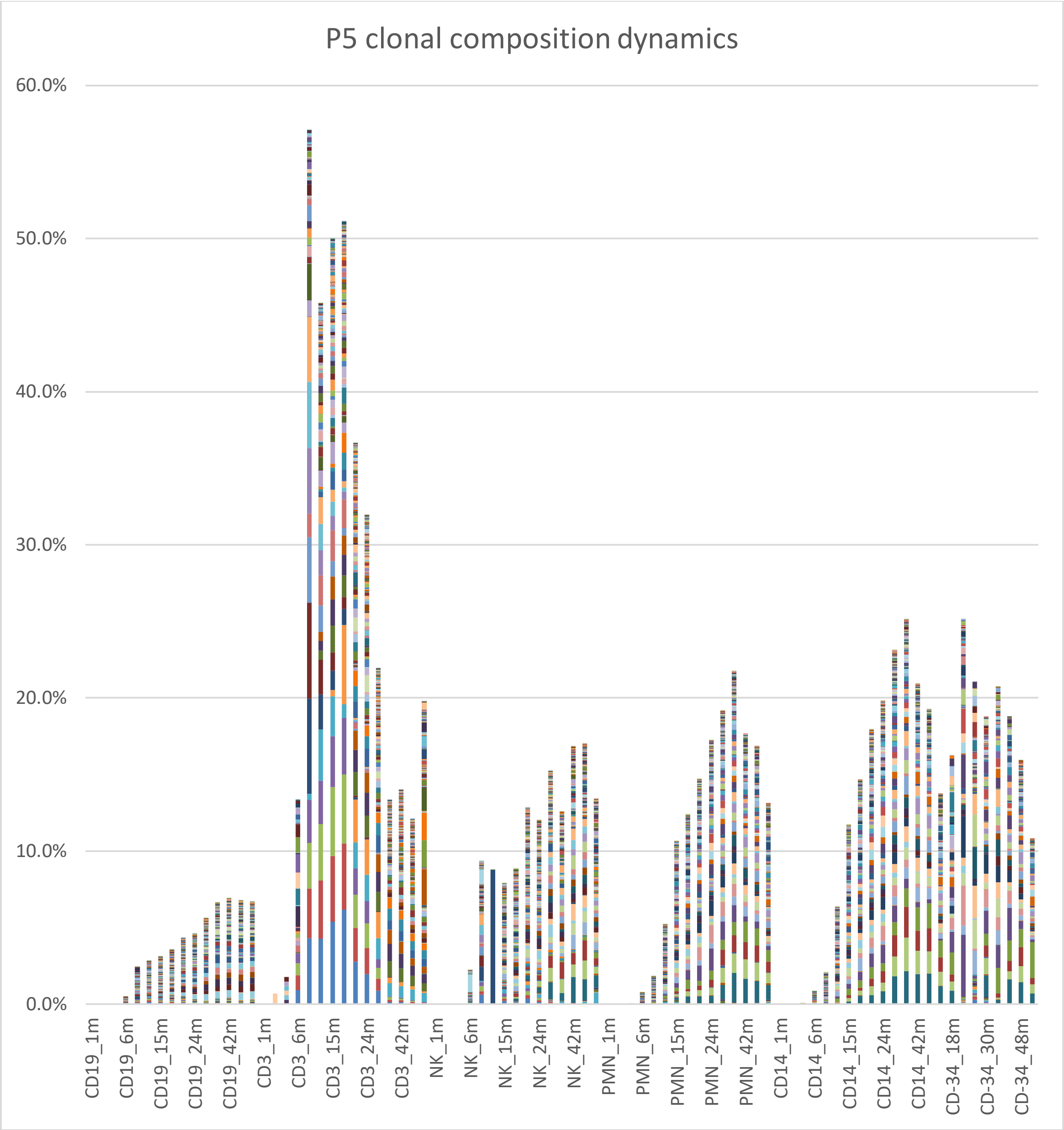
P5 clonal composition dynamics
60.0%
50.0%
40.0%
30.0%
20.0%
10.0%
0.0%
CD19_1m
CD19_6m
CD19_15m
CD19_24m
CD19_42m
CD3_1m
CD3_6m
CD3_15m
CD3_24m
CD3_42m
NK_1m
NK_6m
NK_15m
NK_24m
NK_42m
PMN_1m
PMN_6m
PMN_15m
PMN_24m
PMN_42m
CD14_1m
CD14_6m
CD14_15m
CD14_24m
CD14_42m
CD-34_18m
CD-34_30m
CD-34_48m

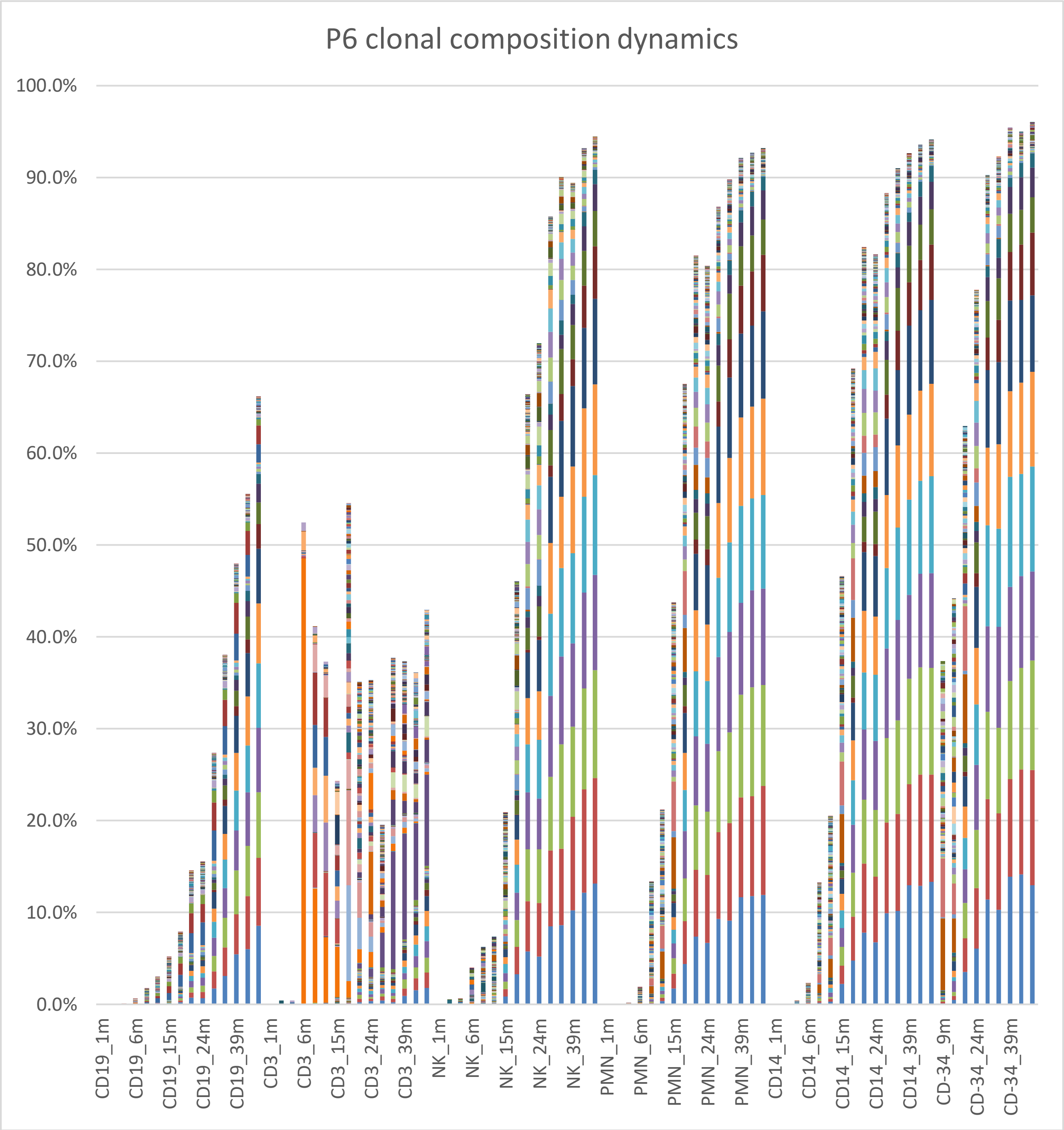
P6 clonal composition dynamics
100.0%
90.0%
80.0%
70.0%
60.0%
50.0%
40.0%
30.0%
20.0%
10.0%
0.0%
CD19_1m
CD19_6m
CD19_15m
CD19_24m
CD19_39m
CD3_1m
CD3_6m
CD3_15m
CD3_24m
CD3_39m
NK_1m
NK_6m
NK_15m
NK_24m
NK_39m
PMN_1m
PMN_6m
PMN_15m
PMN_24m
PMN_39m
CD14_1m
CD14_6m
CD14_15m
CD14_24m
CD14_39m
CD-34_9m
CD-34_24m
CD-34_39m

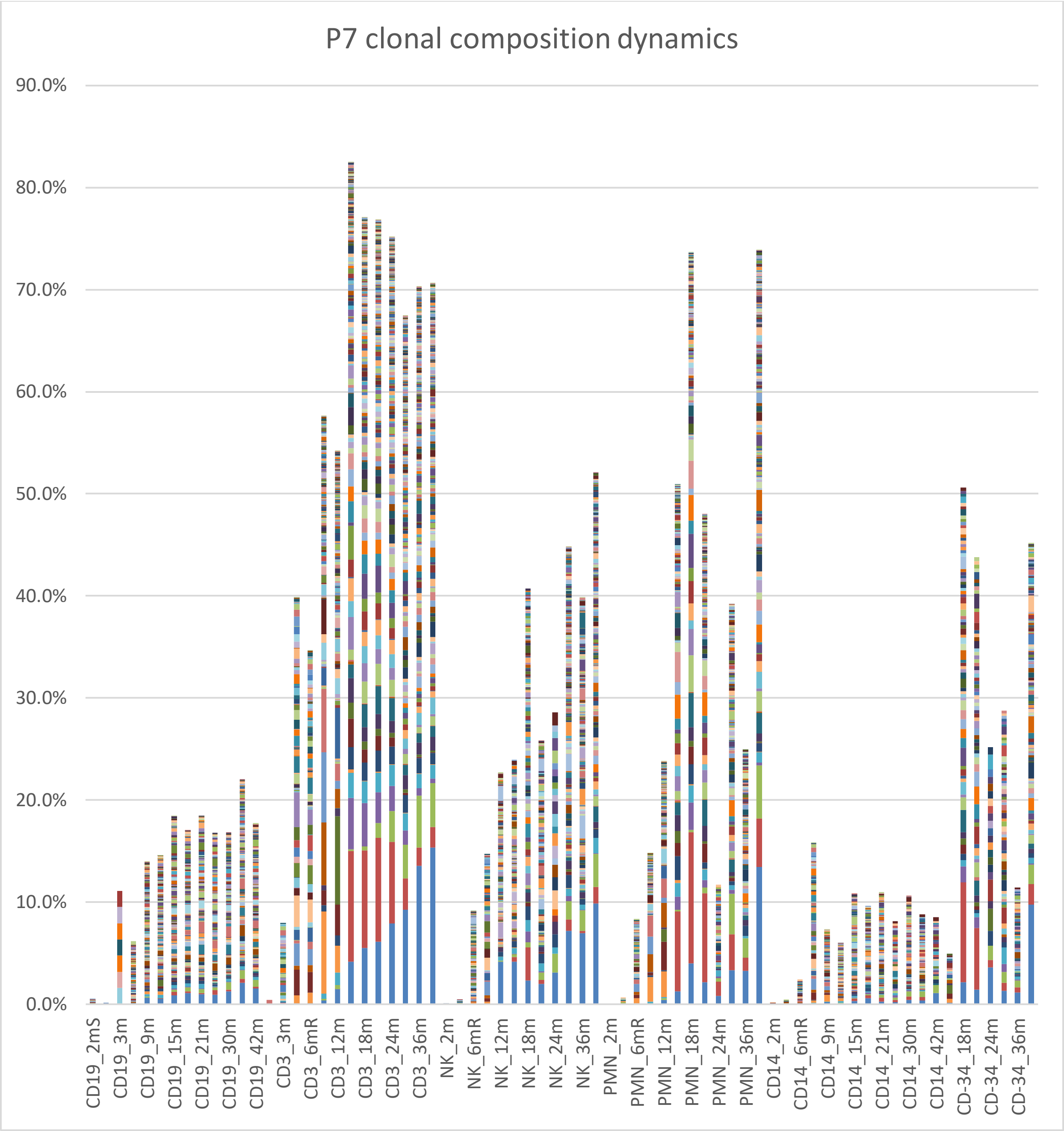
P7 clonal composition dynamics
90.0%
80.0%
70.0%
60.0%
50.0%
40.0%
30.0%
20.0%
10.0%
0.0%
CD19_2mS
CD19_3m
CD19_9m
CD19_15m
CD19_21m
CD19_30m
CD19_42m
CD3_3m
CD3_6mR
CD3_12m
CD3_18m
CD3_24m
CD3_36m
NK_2m
NK_6mR
NK_12m
NK_18m
NK_24m
NK_36m
PMN_2m
PMN_6mR
PMN_12m
PMN_18m
PMN_24m
PMN_36m
CD14_2m
CD14_6mR
CD14_9m
CD14_15m
CD14_21m
CD14_30m
CD14_42m
CD-34_18m
CD-34_24m
CD-34_36m

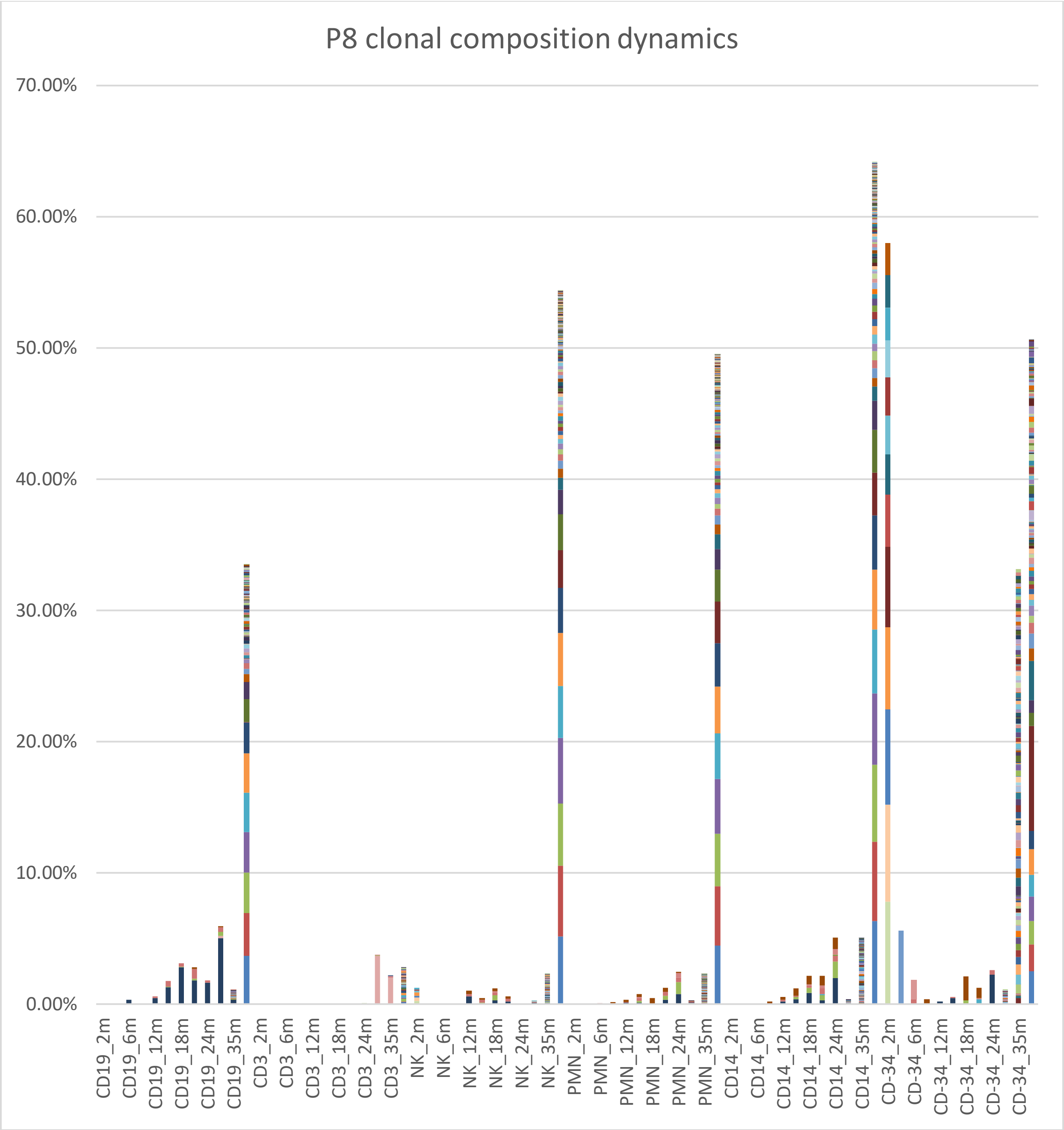

Figure S2 Progression of clonal composition in B, T, NK, PMN, CD14 and CD34 lineages after treatment in subjects P1, P3, P4, P5, P6, P7 and P8.

Figure S3

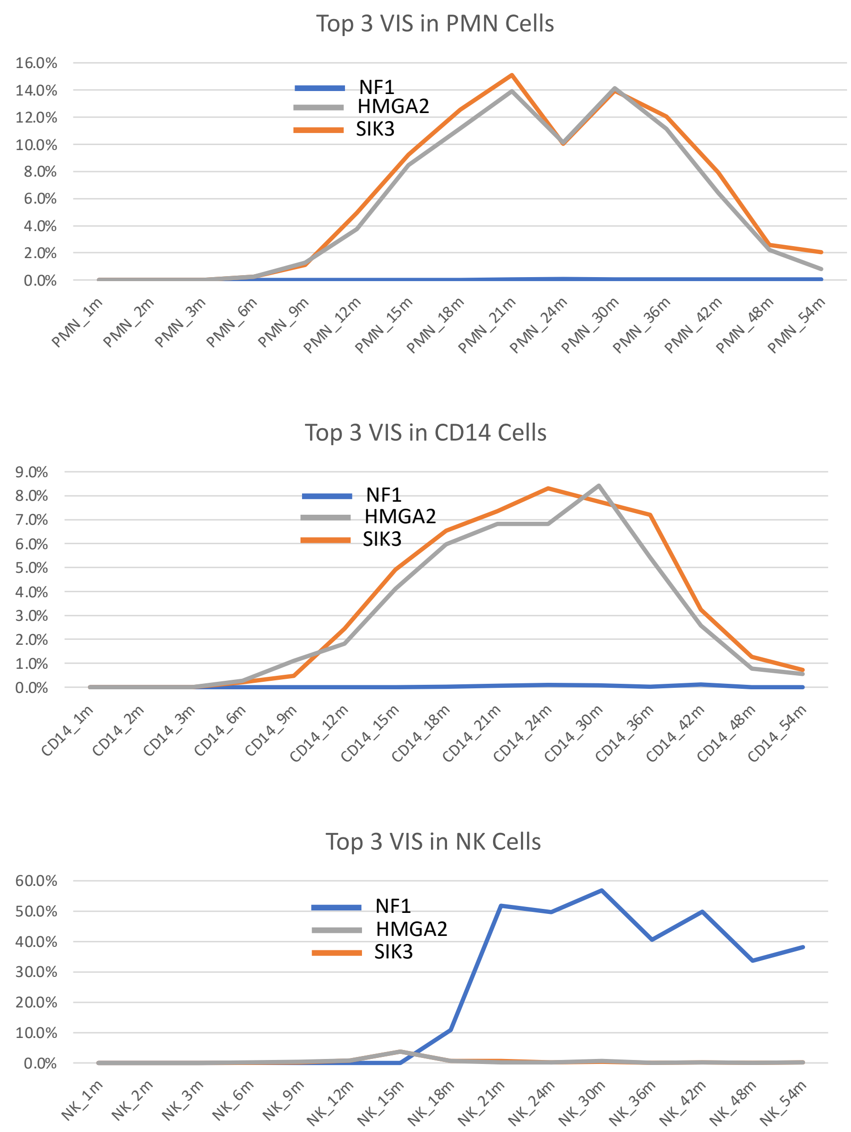

Figure S3 Quantitative frequency assessment of the top three most abundant vector insert sites (VIS) in polymorphonuclear (PMN), CD14 and Natural Killer (NK) cells in P3. Orange curve represents *SIK3*, gray *HMGA2*, blue *NF1*.

Figure S4

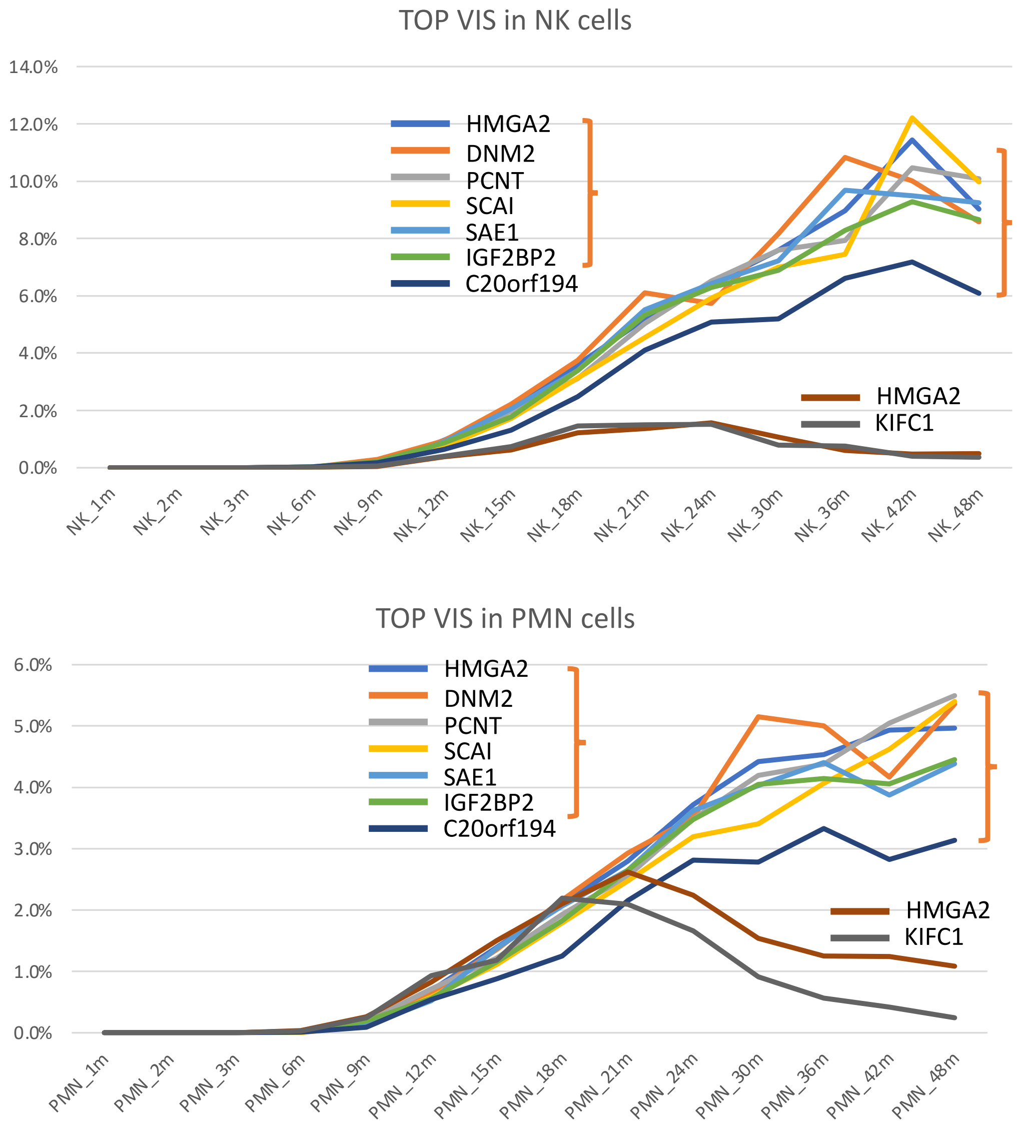

Figure S4 Quantitative frequency assessment of the top most abundant vector insert sites (VIS) in Natural Killer (NK) and polymorphonuclear (PMN) cells in P4.

Figure S5

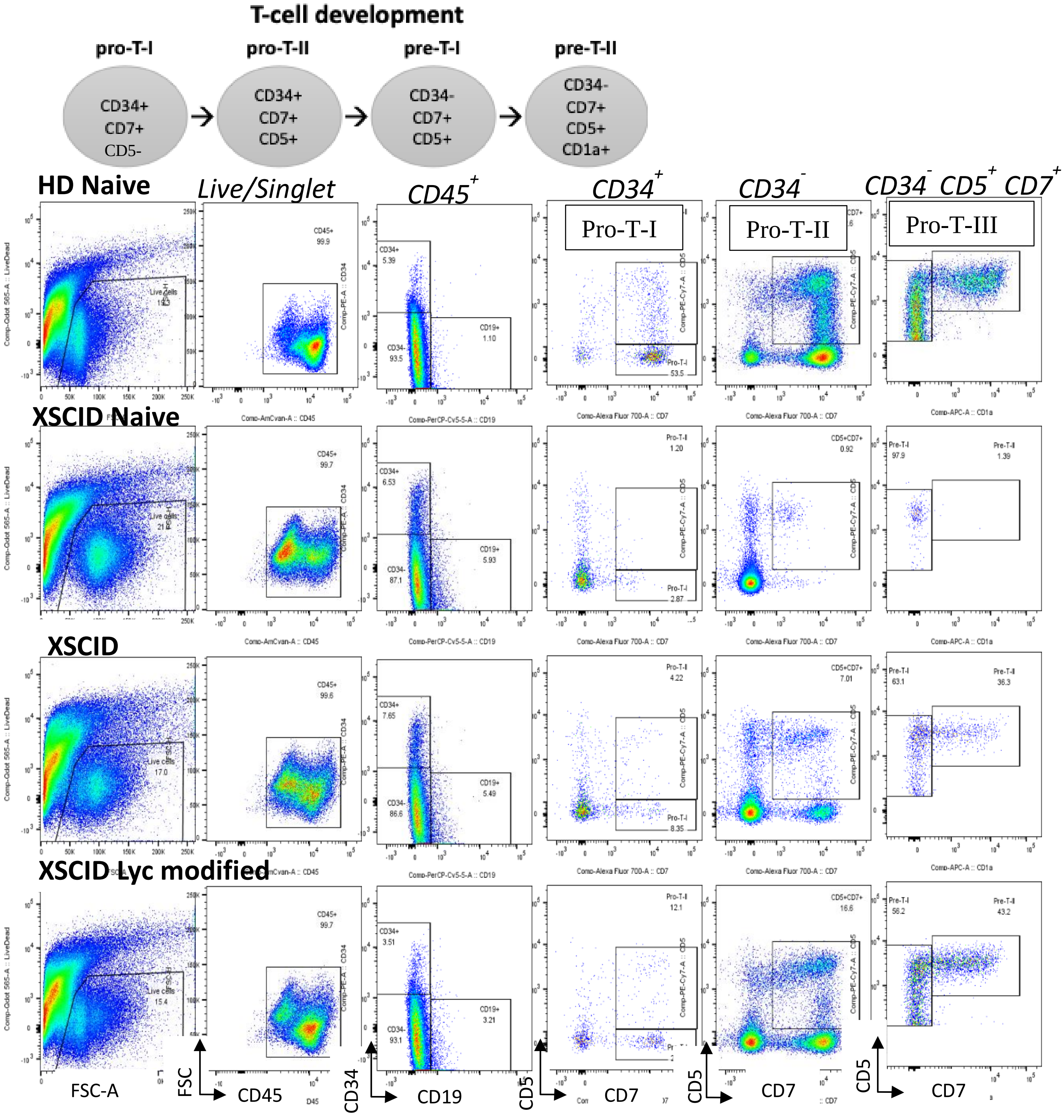

Figure S5

Gating strategy for assessment of T-cell development *in vitro* using Artificial Thymic Organoids. The columns show pro-TI (CD34+CD7+), pro-T-II (CD34+CD7+CD5+), pre-T-I (CD34-CD7+5+), and pre-T-II (CD34-CD7+CD5+CD1a+) populations.
