# Supplementary material for: Lentivector cryptic splicing mediates increase in CD34+ clones expressing truncated HMGA2 in human X-linked severe combined immunodeficiency
Source: Nat Commun. 2022 Jun 28;13:3710. doi: 10.1038/s41467-022-31344-x (PMC9240040; doi:10.1038/s41467-022-31344-x)
Supplement: Supplementary file 2 — Description of Additional Supplementary Files [file 41467_2022_31344_MOESM2_ESM.pdf]

Description of Additional Supplementary Files

File name: Supplementary data 1
Description: Supplementary Data 1 shows top vector integration sites and multicopy clones identified in P1, P3, P4 and P6 by VIS frequency correlation. Detailed information is also provided as Supplemental Data in excel document.

File name: Supplementary data 2
Description: Supplementary Data 2 shows Fusion Transcripts created by vector gene trapping of targeted genes in inducible pluripotent stem clones. VIS that are in the same orientation of gene and inside introns are highlighted and expected to result in premature termination.
