# Supplementary material for: Lentivector cryptic splicing mediates increase in CD34+ clones expressing truncated HMGA2 in human X-linked severe combined immunodeficiency
Source: Nat Commun. 2022 Jun 28;13:3710. doi: 10.1038/s41467-022-31344-x (PMC9240040; doi:10.1038/s41467-022-31344-x)
Supplement: Supplementary file 5 — Reporting Summary [file 41467_2022_31344_MOESM5_ESM.pdf]

nature portfolio

Corresponding author(s): 1. Harry L. Malech, MD
2. Suk See De Ravin, MD, PhD
3. Xiaolin Wu PhD

Last updated by author(s): Mar 15, 2022

Reporting Summary

Nature Portfolio wishes to improve the reproducibility of the work that we publish. This form provides structure for consistency and transparency in reporting. For further information on Nature Portfolio policies, see our Editorial Policies and the Editorial Policy Checklist.

Statistics

For all statistical analyses, confirm that the following items are present in the figure legend, table legend, main text, or Methods section.

n/a	Confirmed	
☐	☒	The exact sample size (*n*) for each experimental group/condition, given as a discrete number and unit of measurement
☐	☒	A statement on whether measurements were taken from distinct samples or whether the same sample was measured repeatedly
☐	☒	The statistical test(s) used AND whether they are one- or two-sided<br>*Only common tests should be described solely by name; describe more complex techniques in the Methods section.*
☒	☐	A description of all covariates tested
☒	☐	A description of any assumptions or corrections, such as tests of normality and adjustment for multiple comparisons
☐	X	A full description of the statistical parameters including central tendency (e.g. means) or other basic estimates (e.g. regression coefficient) AND variation (e.g. standard deviation) or associated estimates of uncertainty (e.g. confidence intervals)
☐	X	For null hypothesis testing, the test statistic (e.g. *F*, *t*, *r*) with confidence intervals, effect sizes, degrees of freedom and *P* value noted<br>*Give P values as exact values whenever suitable.*
☒	☐	For Bayesian analysis, information on the choice of priors and Markov chain Monte Carlo settings
☒	☐	For hierarchical and complex designs, identification of the appropriate level for tests and full reporting of outcomes
☒	☐	Estimates of effect sizes (e.g. Cohen's *d*, Pearson's *r*), indicating how they were calculated

Our web collection on statistics for biologists contains articles on many of the points above.

Software and code

Policy information about availability of computer code

Data collection

BD FACS Diva software v 8.0, QuantaSoft software, ABI Prism 7900HT Sequence detection system, Illumina MiSeq.

Quant tative determination of VCN by ddPCR. To measure the vector-carrying cells, we used a ddPCR (Bio-Rad) assay. The ddPCR assay allows the measurement of absolute copy number without using standard curve. The vector-specific primers and probes are as follows: HIV forward, 5′-CTGTTGTGTGACTCTGGTAACT-3′; HIV reverse, 5′-TTCGCTTTCAAGTCCCTGTT-3′; and HIV probe, 5′-/56-FAM/AAATCTCTA/ZEN/GCAGTGGCGCCCG/3IABkFQ/-3′. We multiplexed a reference gene assay for cell counts in the same reaction [myocardin-like protein 2 (MKL2): forward, 5′-AGATCAGAAGGGTGAGAAGAATG-3′; reverse, 5′-GGATGGTCTGGTAGTTGTAGTG-3′; and probe, 5′-/56-HEX/TGTTCCTGC/ZEN/AACTGCAGATCCTGA/3IABkFQ/-3′]. Cell number was calculated as half of the MKL2 counts because each cell is diploid. VCN was calculated as vectors per cell.
Quantification of clonal expansion of specific integration site. Top expanded clones identified by the VISA sequencing assay were followed up and monitored by specific ddPCR assay. All of our ddPCR assays consisted of a common LTR primer, an LTR probe, and a specific primer for genomic DNA junction (5LTR reverse, 5′-CTGCAGGGATCTTGTCTTCTT-3′; 5LTR junction probe, 5′-/56-FAM/TGGAAGGGC/ZEN/TAATTCACTCCCA/3IABkFQ/-3′; 3LTR forward, 5′-CCCACTGCTTAAGCCTCAATA-3′; 3LTR junction probe, 5′-/56-FAM/AAGTAGTGT/ZEN/GTGCCCGTCTGTTGT/3IABkFQ/-3′). We multiplexed the integration site–specific assay together with the MKL2 reference gene assay, which measured cell counts in the same reaction.

RNAseq. Total RNA was isolated from iPS cell clones. We performed both mRNAseq and total RNAseq using NEB Next Ultra RNA library Prep Kit for Illumina (E7530S) following to manufacturer's recommended protocols (New England Biolabs, Ipswich, MA). The libraries were sequenced with 2x75 bp paired end reads on Illumina NextSeq2000 with P2 reagents (Illumina, San Diego, CA). Data analysis was performed with NextSeq2000 onboard Dragon RNAseq pipeline. For fusion transcript detection with cHS4, fastq files were used to search all reads that contain the cHS4 SA site. The junctions were then split and sorted and mapped to human genome hg38 to find the fusion partners.

Data analysis: Prism GraphPad Software 8.3.0, FlowJoversion 9

For manuscripts utilizing custom algorithms or software that are central to the research but not yet described in published literature, software must be made available to editors and reviewers. We strongly encourage code deposition in a community repository (e.g. GitHub). See the Nature Portfolio guidelines for submitting code & software for further information.

Data

Policy information about availability of data

All manuscripts must include a data availability statement. This statement should provide the following information, where applicable:

- Accession codes, unique identifiers, or web links for publicly available datasets
- A description of any restrictions on data availability
- For clinical datasets or third party data, please ensure that the statement adheres to our policy

Patient integration site data are available upon request. RNAseq data for iPSC clones and P6 patient blood samples are available at NCBI Sequence Read Archive (PRJNA788948: SRR17238208- SRR17238226, which can all be found on the National Center for Biotechnology Information database).

Field-specific reporting

Please select the one below that is the best fit for your research. If you are not sure, read the appropriate sections before making your selection.

☒ Life sciences ☐ Behavioural & social sciences ☐ Ecological, evolutionary & environmental sciences

For a reference copy of the document with all sections, see nature.com/documents/nr-reporting-summary-flat.pdf

Life sciences study design

All studies must disclose on these points even when the disclosure is negative.

Sample size: This is a Phase I/II safety and efficacy study for a rare disease and therefore has a small sample size pre-determined in the clinical protocol. The original statistical design in this protocol was based on the Simon Two-stage design. In this design, it is considered to be unacceptable if the true proportion of patients experiencing immune reconstitution is less than 10% by using the gene therapy approach. On the other hand, it would be considered as promising if this true proportion is 40%. With type one error of 5%, this trial has 80% power for a true proportion of 40% of patients expected to achieve immune reconstitution after 2 years.

Data exclusions: Data from one subject was excluded from long-term clonal analysis because of a fatal pulmonary hemorrhage due to underlying disease at ~2 years following treatment.

Replication: The events observed occurred in multiple human subjects and replication is not relevant.

Randomization: All subjects were monitored for vector insert site analysis for this non-randomized study. Covariates are not controlled and not

Blinding: applicable to this study because they should not affect the primary and secondary objectives of this study.

This is an open label phase I/II safety/efficacy clinical trial that involves chemotherapy prior to cell infusion and blinding is not ethical.

Reporting for specific materials, systems and methods

We require information from authors about some types of materials, experimental systems and methods used in many studies. Here, indicate whether each material, system or method listed is relevant to your study. If you are not sure if a list item applies to your research, read the appropriate section before selecting a response.

Materials & experimental systems

n/a	Involved in the study	
☐	☒	Antibodies
	X	Eukaryotic cell lines
☒	☐	Palaeontology and archaeology
☒	☐	Animals and other organisms
☐	☒	Human research participants
☐	☒	Clinical data
☒	☐	Dual use research of concern

Methods

n/a	Involved in the study	
☒	☐	ChIP-seq
☐	☒	Flow cytometry
☒	☐	MRI-based neuroimaging

Antibodies

Antibodies used: CD14, eBioscience 48-0149-42; CD5 Invitrogen 25-0059-42; CD7 Invitrogen 56-0079-42; CD1a Biolegend 300110; CD34 Biolegend 343506; CD56 Biolegend 304604; CD45 BD Biosciences 560777; CD19 Biolegend 302230; Live/Dead Invitrogen L34959; CD45 BD Biosciences560777;CD3 BD Biosciences56426;CD4 Invitrogen 56-0048-82; CD8a Biolegend301058;TCRabInvitrogen 17-9986-42.

Validation: All primary antibodies are used with appropriate controls per published methods (Bosticardo,M. et al Blood Adv. 2020, 4, 2611-2616.

Human research participants

Policy information about studies involving human research participants

Population characteristics	The human research participants for the clinical study are all male because this is an X-linked disease and the treatment is specific for their underlying X-linked severe combined immunodeficiency.
Recruitment	The subjects are referred by their local immunologists and transplanters. The bias is towards subjects with significant clinical complications but the bias in participating subjects' clinical manifestations is not likely to affect the clonal expansions reported in this study.
Ethics oversight	NIH Institutional Regulatory Board, NHLBI Data Safety and Monitoring Board

Note that full information on the approval of the study protocol must also be provided in the manuscript.

Clinical data

Policy information about clinical studies

All manuscripts should comply with the ICMJE guidelines for publication of clinical research and a completed CONSORT checklist must be included with all submissions.

Clinical trial registration	ClinicalTrials.gov ID NCT01306019
Study protocol	NIAID clinical protocol #11-I-0007
Data collection	The Clinical Center, National Institutes of Health, from 2011 to 2021
Outcomes	The primary objective is to assess the efficacy of immune reconstitution in XSCID patients transplanted with autologous CD34+ HSC that have been transduced with a self-inactivating lentiviral vector expressing a functional γc gene. Laboratory and clinical assessments will be performed monthly for the first 3 months and then every 3 months until the primary endpoint evaluation at 2 years. Since we expect there to be a lag in the demonstration of clinical benefits from any improvement of immunity. Therefore, the primary study endpoint evaluation for the efficacy (laboratory and clinical) will be at 2 years after treatment. Evaluation will include assessment of general clinical benefit, T-cell function, and B-cell reconstitution.<br><br>The secondary endpoints are:<br>.To determine the incidence of serious side effects due to lentiviral gene transfer out to at least 5 years after treatment, though periodic follow-up will continue for 15 years as per FDA Guidance<br>-To determine the integration site distribution of the lentiviral vector in reconstituted peripheral blood cells at intervals out to at least 5 years after treatment.

Flow Cytometry

Plots

Confirm that:

☒ The axis labels state the marker and fluorochrome used (e.g. CD4-FITC).

☒ The axis scales are clearly visible. Include numbers along axes only for bottom left plot of group (a 'group' is an analysis of identical markers).

☒ All plots are contour plots with outliers or pseudocolor plots.

☒ A numerical value for number of cells or percentage (with statistics) is provided.

Methodology

Sample preparation	Cells were washed in FACS buffer (PBS with 0.1% bovine serum albumin) and centrifuged for 5 minutes at 1800 rpm. For cell surface staining, cells were incubated for 30 minutes in the dark with antibodies and washed in FACS buffer
Instrument	Flow cytometry analysis was performed using a BD Canto flow cytometer.
Software	DIVA software (BD Biosciences) and FlowJo analysis software (Tree Star).
Cell population abundance	The distinct cell populations were distinguished by cell surface markers but were not isolated by sorting.
Gating strategy	Starting from live cells from Forward/side scatter, the cells are then gated on CD34+ versus SSC-H, of the CD34+ cells,pro-T-I and pro-T-II cells are gated based on CD7 versus CD5, and CD34- cells are gated into CD7+CD5+, followed by CD1a neg (pre-T-I) and CD1a+ (pre-T-II) cells for T-cell development (Bosticardo, M. Blood Adv. 2020 Jun 23;4(12):2611-2616.)<br><br>The comparison is made between healthy donor CD34 cells, XSCID patient naive CD34 and lentivector transduced CD34 cells.

☒ Tick this box to confirm that a figure exemplifying the gating strategy is provided in the Supplementary Information.
